# Mutations in *CNNM4* Cause Jalili Syndrome, Consisting of Autosomal-Recessive Cone-Rod Dystrophy and Amelogenesis Imperfecta

**DOI:** 10.1016/j.ajhg.2009.01.009

**Published:** 2009-02-13

**Authors:** David A. Parry, Alan J. Mighell, Walid El-Sayed, Roger C. Shore, Ismail K. Jalili, Hélène Dollfus, Agnes Bloch-Zupan, Roman Carlos, Ian M. Carr, Louise M. Downey, Katharine M. Blain, David C. Mansfield, Mehdi Shahrabi, Mansour Heidari, Parissa Aref, Mohsen Abbasi, Michel Michaelides, Anthony T. Moore, Jennifer Kirkham, Chris F. Inglehearn

**Affiliations:** 1 Leeds Institute of Molecular Medicine, University of Leeds, St. James's University Hospital, Leeds LS9 7TF, UK; 2 Leeds Dental Institute, University of Leeds, Leeds LS2 9LU, UK; 3 AVENIR Inserm, Université de Strasbourg, 67085 Strasbourg, France; 4 Centre de référence pour les affections ophtalmologiques héréditaires (CARGO), Hôpitaux Universitaires de Strasbourg, 67000 Strasbourg, France; 5 Department of Paediatric Dentistry, Faculty of Dentistry, Louis Pasteur University, 67000 Strasbourg, France; 6 Reference Centre for Oral Manifestations of Rare Diseases, Service de Soins Bucco-Dentaires, Hôpitaux Universitaires de Strasbourg, 67000 Strasbourg, France; 7 Institute of Genetics and Molecular and Cellular Biology, Inserm, U596, CNRS, UMR7104, 67404 Illkirch Cedex, France; 8 Centro Clinico de Cabeza y Cuello, Guatemala City 01010, Guatemala; 9 Hull and East Yorkshire Eye Hospital, Hull HU3 2JZ, UK; 10 Child Dental Health, Dundee Dental Hospital, Dundee DD1 4HR, UK; 11 Department of Ophthalmology, Inverclyde Royal Hospital, Greenock PA16 0XN, UK; 12 Faculty of Dentistry, Tehran University of Medical Sciences, 1417613151 Tehran, Iran; 13 Department of Medical Genetics, Tehran University of Medical Sciences, 1417613151 Tehran, Iran; 14 Institute of Ophthalmology, University College London, London EC1V 9EL, UK; 15 Moorfields Eye Hospital, London EC1V 2PD, UK

## Abstract

The combination of recessively inherited cone-rod dystrophy (CRD) and amelogenesis imperfecta (AI) was first reported by Jalili and Smith in 1988 in a family subsequently linked to a locus on chromosome 2q11, and it has since been reported in a second small family. We have identified five further ethnically diverse families cosegregating CRD and AI. Phenotypic characterization of teeth and visual function in the published and new families reveals a consistent syndrome in all seven families, and all link or are consistent with linkage to 2q11, confirming the existence of a genetically homogenous condition that we now propose to call Jalili syndrome. Using a positional-candidate approach, we have identified mutations in the *CNNM4* gene, encoding a putative metal transporter, accounting for the condition in all seven families. Nine mutations are described in all, three missense, three terminations, two large deletions, and a single base insertion. We confirmed expression of Cnnm4 in the neural retina and in ameloblasts in the developing tooth, suggesting a hitherto unknown connection between tooth biomineralization and retinal function. The identification of *CNNM4* as the causative gene for Jalili syndrome, characterized by syndromic CRD with AI, has the potential to provide new insights into the roles of metal transport in visual function and biomineralization.

## Main Text

Cone-rod dystrophy (CRD [MIM 120970]) usually manifests in childhood or early adulthood with predominant or equal loss of cone compared to rod photoreceptors, reduced visual acuity, color-vision abnormalities, photophobia, and visual-field loss.^1^ Mutations in *ABCA4* (MIM^∗^601691), *AIPL1* (MIM ^∗^604392), *CRX* (MIM +602225), *GUCA1A* (MIM ^∗^600364), *GUCY2D* (MIM ^∗^600179), *PITPNM3* (MIM ^∗^608921), *RIMS1* (MIM ^∗^606629), SEM*A4A* (MIM ^∗^6072920), *RPGR* (MIM ^∗^312610), *PROM1* (MIM ^∗^604365), and *UNC119* (MIM ^∗^604011) have been associated with CRD, which can be inherited in an autosomal-dominant, autosomal-recessive, or X-linked manner (RetNet). These genes encode proteins of diverse functions, including participants in the phototransduction cascade and the visual cycle, structural components of photoreceptors, and photoreceptor-specific transcription factors.

In comparison with retinal degeneration, little is understood about the genetic causes of abnormal tooth biomineralization (reviewed by Bailleul-Forestier and colleagues^2^, ^3^). This group of conditions is traditionally considered to involve either enamel (amelogenesis imperfecta [AI] [MIM #204700]) or dentine (dentinogenesis imperfecta [DI] [MIM #125490] and dentine dysplasias [MIM %125400]), the two major hard-tissue components of teeth. Nonsyndromic AI has been attributed to mutations in five genes, which fall into three groups and involve the following: enamel-matrix proteins (*AMELX*^4^ [MIM ^∗^300391]; *ENAM*^5^ [MIM ^∗^606585]), enzymes controlling postsecretory processing of enamel-matrix proteins (*KLK4*^6^ [MIM ^∗^603767]; *MMP20*^7^ [MIM ^∗^604629]), and a gene of unknown function (*FAM83H*^8^ [MIM ^∗^611927]). Only a single gene (*DSPP* [MIM ^∗^125485]) has been implicated in nonsyndromic DI.^9^

In addition, CRD, AI, or DI can be part of syndromes involving multiple tissues and organs. CRD is a feature of some forms of Bardet Biedl syndrome (MIM 209900), Alstrom syndrome (MIM 203800), spinocerebellar ataxia type 7 (MIM 164500), and selected syndromes characterized by ectodermal abnormalities. AI and DI have been described as part of syndromes involving bone abnormalities, giving insight into different forms of human biomineralization.^3^, ^10^ However, there is no agreed term for developmental abnormalities of enamel plus dentine, even though this condition is increasingly recognized. The abnormal dental phenotypes observed with *MSX2* mutations have been described as AI even though there is evidence of dentine involvement.^11^

The combination of familial CRD and AI (MIM %217080) was first reported in a large consanguineous Arab kindred living in the Gaza strip (Gaza A),^12^ and linkage to chromosome 2q11 was reported.^13^ The subsequent description of an affected sib pair from Kosovo that had a similar clinical phenotype and that was consistent with linkage to 2q11 suggested that the combination of CRD and AI might exist as a genetically homogenous syndrome.^14^ Descriptions of the ocular phenotype, including assessment of the visual impairment, were comprehensive in these reports, but insight into the tooth phenotype was more limited. Both cases showed an autosomal-recessive pattern of inheritance.

We identified a further five ethnically diverse unrelated families sharing the distinctive combination of recessively inherited infancy-onset CRD and dental biomineralization defects with grossly abnormal enamel. These include families from Turkey, Guatemala, Iran, and Scotland and a second family from Gaza of probable Jordanian origin (Gaza B), listed in Table 1 together with the original Gaza family (Gaza A) and the Kosovan sib pair. Family members from all seven pedigrees were recruited to this study and subjected to ophthalmic and dental examination. The study of human subjects was performed with informed patient consent according to the principles of the Declaration of Helsinki, with a process approved by a UK Ethics committee. Genomic DNA was extracted from peripheral blood lymphocytes by standard protocols, and teeth were also obtained where available.

**Table 1:** A Summary of the *CNNM4* Mutations Identified

Origin	Reference	Family History of Consanguinity	Mutation 1	Mutation 2
Gaza A	12, 13	Yes	c.599C→A; Ser200Tyr	c.599C→A; Ser200Tyr
Kosovo	14	No	c.1312 dupC; Leu438ProfsX9	c.1312 dupC; Leu438ProfsX9
Gaza B		Yes	c.1813 C→T; Arg605X	c.1813 C→T; Arg605X
Guatemala		No	c.2149C→T; Gln717X	c.62_145 del; Leu21HisfsX185
Turkey		Yes	c.586T→C; Ser196Pro	c.586T→C; Ser196Pro
Iran		Yes	c.1-?_1403+?del	c.1-?_1403+?del
Scotland		No	c.971T→C; Leu324Pro	c.1690C→T; Gln564X

**Figure 1:**
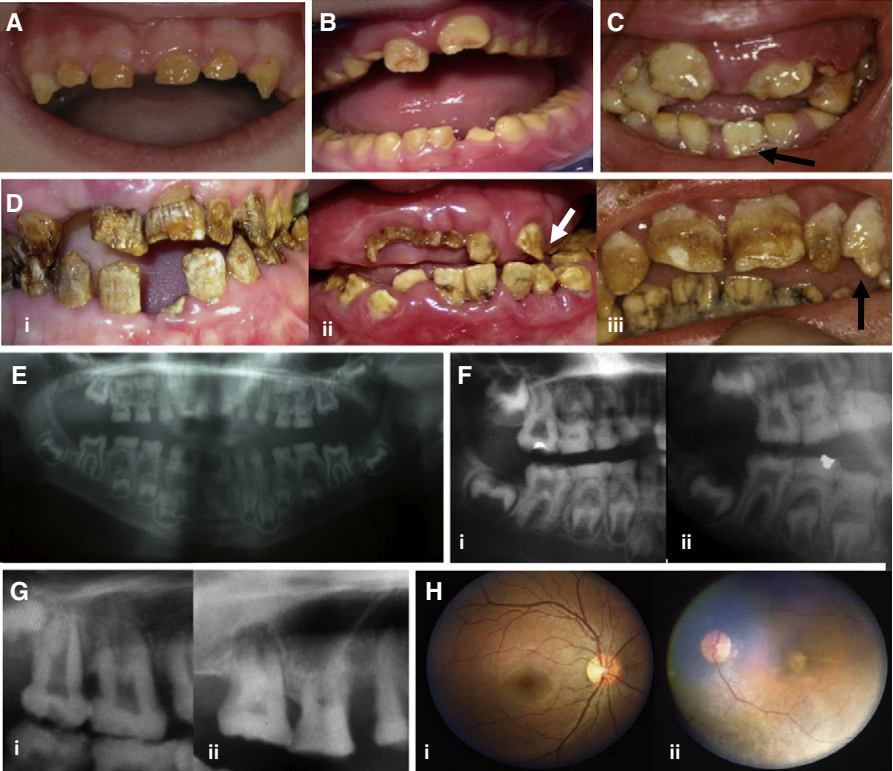
Clinical Features of Jalili Syndrome (A–D) Dentition. (A) Turkish family: Deciduous teeth in a 5-year-old are small, microdont, yellow, and almost devoid of enamel. (B) Scottish family: Gross attrition of occlusal surfaces of the primary molar teeth by age 6 years, in the absence of gross enamel loss from nonocclusal surfaces. The labial surfaces of the erupting upper permanent central incisors are irregular, with early posteruptive enamel loss. (C) Gaza B family: Some teeth at the time of eruption, such as the lower-left permanent incisor illustrated in a 6-year-old, had a near-normal crown morphology and were a paler cream color compared to that of teeth present in the mouth for much longer. (D) (i) Iranian family: By age 24 years, virtually no enamel remains. (ii) Guatemalan family: Lower permanent incisors have mild surface pitting and enamel surface crazing, yet they otherwise have relatively normal morphology compared to that of the more recently erupted upper-left permanent canine tooth (indicated by arrow) that is virtually devoid of enamel in this 12-year-old. Coronal aspects of permanent upper-central incisor teeth are almost completely lost. (iii) Gaza A family: Lower permanent incisors are relatively spared compared to upper teeth. Upper permanent incisors are pitted and irregular, whereas the upper-left permanent canine (indicated by the arrow) is virtually devoid of enamel. (E–G) Dental radiographs. The expected distinct contrast between the comparatively more radiodense enamel and dentine is absent in primary and secondary teeth, which limits insight into the proportions of enamel to dentine. (E) Turkish family 5-year-old: There is diminished enamel volume involving all erupted teeth due to posteruptive loss related to enamel hypomineralization and variable hypoplasia. Permanent teeth are taurodont, especially the upper first molar teeth, characterized by bulbous crowns, large pulp chambers, and thinner roots than expected. The deciduous molar teeth, which exhibit significant occlusal attrition, have pulp chambers that are largely radio-opaque, which is likely to represent dentine. (F) (i) Turkish family 6-year-old and (ii) Scottish family 6-year-old. Similar features are observed in erupted deciduous molar and permanent first molar teeth. The forming, unerupted lower permanent premolar and second molar crowns have near-normal morphology with some surface irregularities. (G) (i) Guatemalan family 12-year-old: Pulp chambers and root canals in permanent teeth are initially obvious, particularly in taurodont teeth. (ii) Gaza A family adult: Pulp chamber and root canals diminish in size with time in teeth that exhibit posteruptive loss of coronal tissue including occlusal attrition. (H) Fundus examination: Gaza A family. Varying degrees of macular atrophy were observed in all affected individuals as illustrated by two adults, (i) and (ii), the latter being more extreme. These findings were consistent with those previously published.^14^

Phenotypic characterization demonstrated consistent dental and ocular phenotypes between families^12^, ^13^, ^14^ (Figure 1). In all affected members of these pedigrees, the enamel of the primary and secondary dentitions was grossly abnormal and prone to rapid posteruptive failure, in part reflecting hypomineralization. The consistent presence of taurodont permanent molar teeth implicated a coexisting abnormality of morphology involving dentine. Significant visual impairment was evident in all affected individuals in infancy or early childhood, with progressive loss of vision with advancing age. Nystagmus was observed as early as the first few months of life and often represented the first clinical sign of abnormal vision. In all affected individuals, there was early and progressive loss of visual acuity, with worsening central vision followed by failure of peripheral vision. There was marked photophobia. A marked impairment of color vision was apparent early in life. Electroretinography (ERG) was characterized by early loss of cone and rod function, with cones being more severely affected. There was evidence of further deterioration in the ERG in one individual retested to the same protocol at ages 3 and 11 years. All ERGs were consistent with those previously reported for the Kosovan family.^14^

To determine whether this was indeed a homogeneous syndrome, we undertook genetic analyses in these families. Although the Scottish family consists of a single case and was too small for genetic analysis, Affymetrix 50K SNP array analysis in the Turkish family (data not shown) and microsatellite genotyping in the remaining three new families (Tables S2–S4 available online) revealed linkage to or homozygosity at 2q11, confirming the existence of a genetically homogenous CRD and AI syndrome, which we now propose to name Jalili syndrome. Genotyping by Affymetrix Genome-Wide Human SNP Array 6.0 in the Turkish, Guatemalan, Scottish, and Gaza B families, as well as in the original Gaza family and the Kosovo sib pair, failed to reveal large deletions that would point to a contiguous gene-deletion syndrome. However, this analysis confirmed homozygous regions at 2q11 in the known consanguineous families and in the Kosovan family, which was not previously known to be consanguineous, refining the locus to a 10.6 Mb region between RS2628473 and RS1901284 (Table S5) containing 71 genes. High-resolution microsatellite mapping in the Iranian family also supported the existence of a homozygous linked region on 2q11.

**Figure 2:**
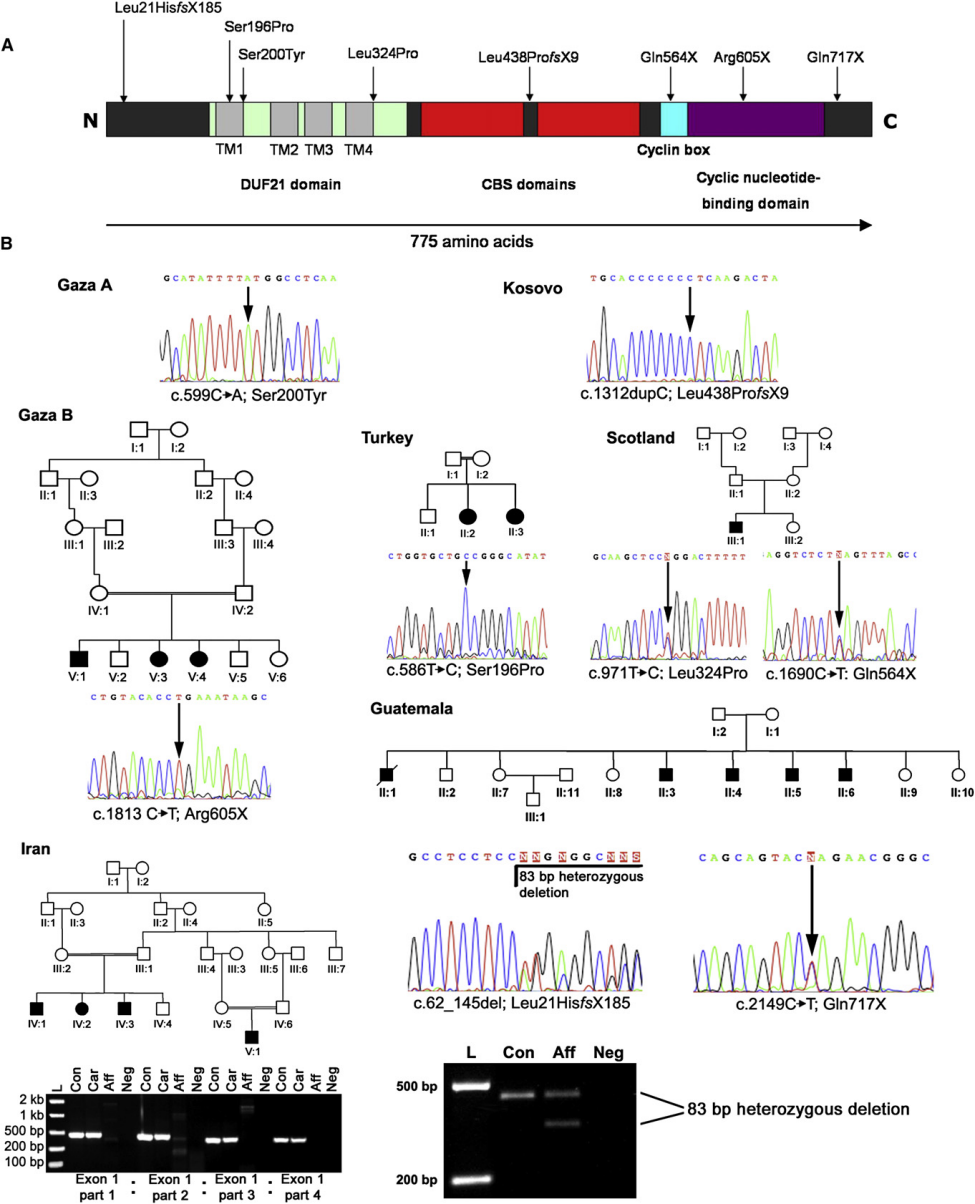
*CNNM4* Mutations and Jalili Syndrome Family Pedigrees (A) Schematic of CNNM4 including protein domains and mutations discovered in affected members of Jalili syndrome families. The following abbreviations are used: CBS domain, cystathionine beta-synthase, core domain; DUF21 domain, domain of unknown function DUF21; TM, transmembrane helix. (B) Sequence of mutations with pedigrees of newly identified families. The Gaza A and Kosovo families have been described in detail elsewhere.^12^, ^13^, ^14^ PCR products generated from members of the Guatemalan and Iranian families demonstrate a heterozygous deletion in the former and a homozygous complete deletion of exon 1 in affected members of the latter by agarose gel electrophoresis. In the Iranian family, heterozygous carriers are indistinguishable from controls, given that the PCR is not quantitative. The following abbreviations are used: L, DNA ladder; Con, control individual DNA; Aff, affected individual DNA; Car, carrier individual DNA; Neg, negative control.

Concluding that Jalili syndrome was likely to be caused by mutations in a single gene, we sequenced 11 candidate genes in the 2q11 region and identified deleterious mutations in the *CNNM4* gene (MIM ^∗^607805) (Table 1 and Figure 2). Nine mutations are described in all, three missense changes, three termination mutations, two large deletions, and a single base insertion. Each mutation cosegregated consistently with the disease phenotype, and the three missense mutations effect nonconservative changes in highly conserved residues of the protein. Mutations in Turkish, Kosovan, Iranian, and both Gaza families were excluded in 112 DNAs from Jordanian control individuals, whereas mutations in the Guatemalan and Scottish families were excluded in 96 DNAs from control individuals of European descent. Point mutations were excluded by direct sequencing and deletions by PCR and size fractionation on an agarose gel.

**Figure 3:**
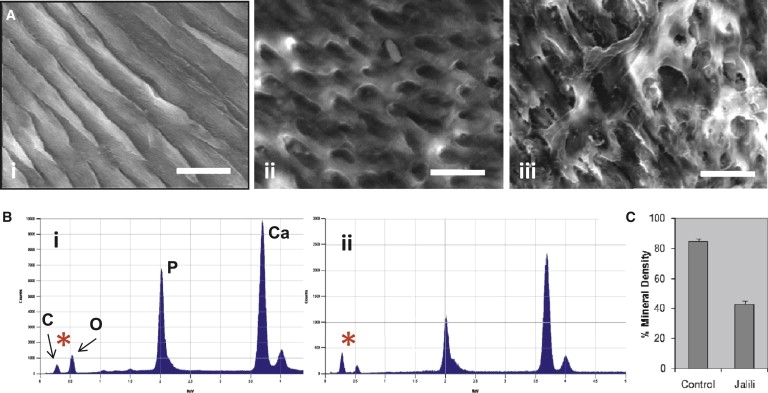
Laboratory Phenotyping of Teeth Standard methods were used for tooth-section (100 μm) preparation from Jalili syndrome teeth and normal controls for subsequent scanning electron microscopy (SEM), transverse microradiography (TMR), and energy dispersive X-ray spectroscopy (EDX).^15^, ^37^ (A) SEM: The dense, ordered rod structure of control enamel in which individual constituent hydroxyapatite crystals making up the rods can be discerned (i) contrasts with that observed in Jalili syndrome, which is characterized by incompletely formed, irregular enamel rods with detailed structure obscured by amorphous material (ii) and focal areas of more disordered enamel where the amorphous covering material is concentrated into larger, coherent areas (iii). The bar represents 10 μm. (B) EDX spectra were examined specifically for investigating the spectra for carbon (C) and oxygen (O), rather than for those representing calcium (Ca) and phosphorous (P). The ratio of the two most left-hand peaks, C and O (^∗^), are reversed in control and affected teeth. In control enamel (i), the C:O ratio is low compared to that in Jalili Syndrome enamel (ii). This is consistent with inappropriately raised amounts of organic material in Jalili syndrome enamel. (C) Histogram illustrating the results of the TMR. The mean mineral percentage in Jalili syndrome teeth is 43.1% ± 1.7% (n = 5) compared to a mean for the control teeth of 84.6% ± 1.5% (n = 5). The difference between these two values is statistically significant (p < 0.001) (Student's t test). Error bars represent the standard deviation.

To characterize the tooth phenotype in greater detail, we obtained two exfoliated deciduous molar teeth from the affected individual from the Scottish family, together with matched control teeth. These were sectioned longitudinally and then subjected to ultrastructural analysis (Figure 3). This analysis demonstrated that affected enamel was mineralized only to approximately 50% of the value for normal enamel and was similar to that for dentine. The resulting radiographic appearance is similar to that seen in hypomaturation AI resulting from *MMP20* and *KLK4* mutations.^6^, ^7^ In addition, the microstructure of the enamel prisms in the Jalili syndrome teeth was obscured by an amorphous organic material. This appearance of the affected enamel was in many ways similar to that published previously for teeth clinically diagnosed as affected by hypomaturation AI.^15^

**Figure 4:**
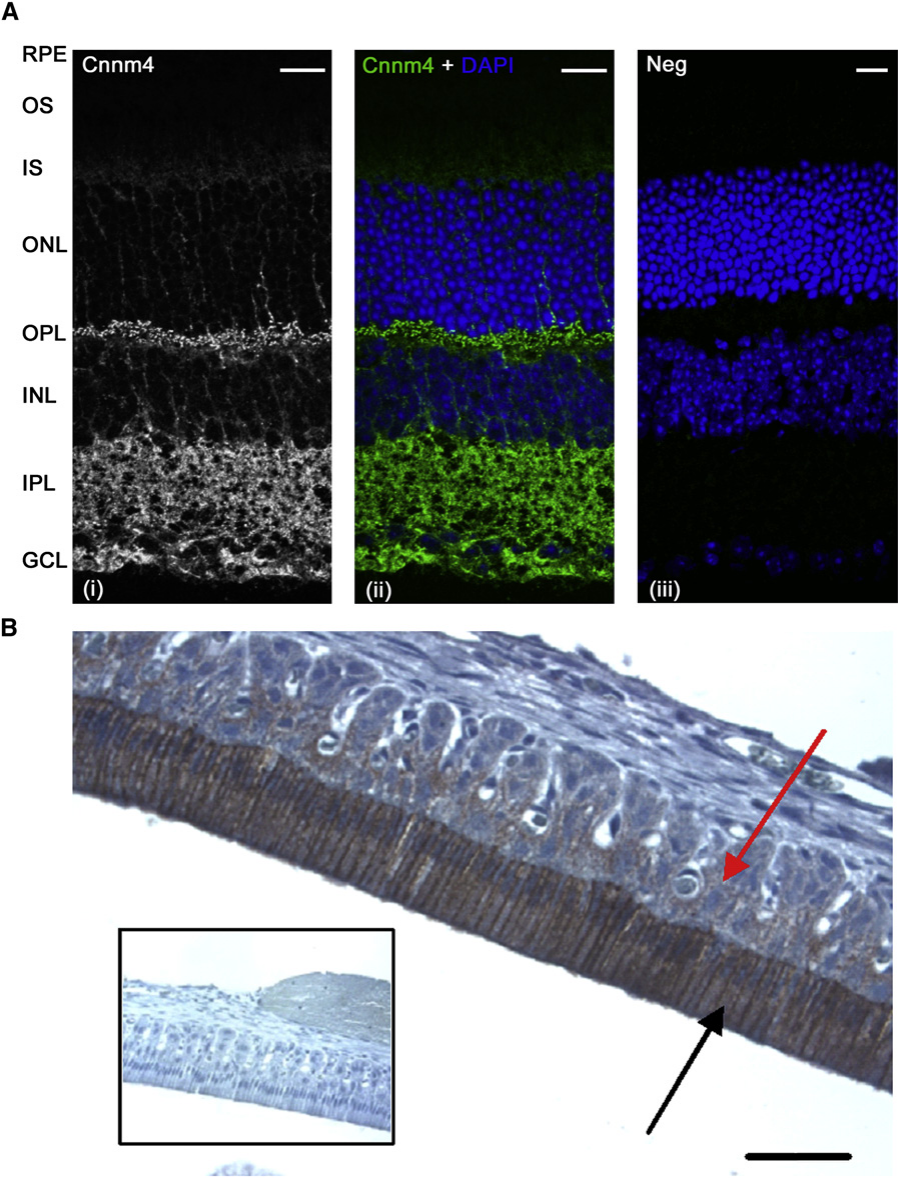
Localization of Cnnm4 in Mouse Retina and Rat Jaw (A) Mouse eye cryosections were prepared by standard methods and incubated with either a rabbit polyclonal antibody to CNNM4 (Atlas Antibodies AB, HPA017732) or rabbit IgG isotype negative control (Invitrogen). Bound primary antibody was detected with Alexa Fluor 488 conjugated secondary antibody. Cnnm4 immunoreactivity is observed in the neural retina, with highest expression in the outer plexiform layer (OPL), inner plexiform layer (IPL), and ganglion cell layer (GCL), with nucleus position identified by DAPI counterstain. The following abbreviations are used: RPE, retinal pigment epithelium; OS, photoreceptor outer segments; IS, photoreceptor inner segments; ONL, outer nuclear layer; INL, inner nuclear layer. Scale bars represent 20 μm. (B) Paraffin-embedded sections (5 μm) of demineralized rat mandible including the central incisor tooth were prepared by standard methods and incubated with the CNNM4 antibody. Bound primary antibody was detected with EnVision (Dako). Cnnm4 immunoreactivity is observed in the enamel organ, particularly in the ameloblasts (indicated by the black arrow), which are heavily labeled during transition and maturation (labeling is absent from ameloblasts in the secretion phase). Comparatively less expression is evident in the remaining elements of the enamel organ (indicated by the red arrow). Odontoblasts and the subodontoblastic zone demonstrate some, but much less intense, labeling (data not shown). Inset shows negative control in which primary antibody has been omitted. The bar represents 50 μm.

In addition, we performed immunofluorescence on mouse retina sections and immunohistochemistry on rat incisors and confirmed Cnnm4 expression in both retina and developing teeth (Figure 4). Ameloblasts were heavily labeled during the transition and maturation phases of amelogenesis, but labeling was absent from ameloblasts during the secretion phase. Odontoblasts, which secrete dentine matrix, were also labeled but with reduced intensity. The observation of Cnnm4 immunoreactivity in rat incisor ameloblasts is consistent with involvement in enamel formation and appears to be membrane associated, consistent with the predicted four-transmembrane-domain structure of the protein. In the retina, Cnnm4 was detected in the inner segments, in agreement with published data confirming presence of Cnnm4 in the photoreceptor inner segment by mass spectrometry,^16^ but it was predominantly localized to the outer plexiform layer (OPL), inner plexiform layer (IPL), and ganglion cell layer (GCL), containing the axons, dendrites, and synaptic terminals of the neuronal cells of the retina.

*CNNM4* was originally identified as a member of a gene family with four protein-encoding members in humans, each sharing a 31 residue repeat present in cyclin boxes.^17^ However, these proteins do not appear to have cyclin function in vivo. CNNM1 appears to act as a cytosolic copper chaperone,^18^ whereas the family member most closely related to CNNM4, CNNM2 (68.1% similarity and 56.5%, homology), has been identified as a magnesium transporter.^19^ Guo et al. used metal toxicity assays in HEK293 cells expressing CNNM4 to implicate the protein in metal transport, and by using a yeast two-hybrid system, they suggested an interaction with the metal chaperone COX11.^20^ However, it is difficult to determine the significance of the interaction with COX11, which has been shown to be a component of the mitochondrial inner membrane.^21^ It is possible to envisage a role for CNNM4 in metal transport in biomineralization, wherein the protein could remove unwanted ions, such as magnesium, that are known to interfere with crystal formation in enamel^22^ from the developing enamel matrix. Indeed, magnesium has been detected in relatively high concentrations in the earliest secreted enamel, with decreasing concentrations toward the enamel surface and increased concentrations in hypomineralized enamel.^23^, ^24^ The immunolocalization of Cnnm4 to ameloblast cell membranes is consistent with such a function. The retained amorphous organic material seen in the affected teeth may be a result of increased protein binding to the altered crystal surfaces and subsequent hindrance of the normal proteolytic breakdown of the enamel matrix. This retained protein would then, in turn, inhibit the further crystal growth that normally occurs and results in enamel maturation. The unusual physiological conditions of the retina, in which cells such as the photoreceptors exist in a depolarized state in the absence of stimulus and have an exceptionally high cellular metabolism, may also present an unusual need for specialized Mg^2+^ or other metal transport. Mg^2+^ in particular is necessary for many cellular functions, including its role as a cofactor for enzymes of the phototransduction cascade, and global magnesium deficiency has been shown to have severe effects on the retina, leading to photoreceptor and retina pigment epithelium cell death.^25^ Prominent staining of Cnnm4 in the OPL, IPL, and GCL suggests a possible role in the maintenance of the ionic content of the extracellular space around the axons, dendrites, and synapses in the neural retina. It is interesting to note that NMDA receptors and GABA receptors have been detected in these layers of the retina (for reviews, see Shen et al. and Yang^26^, ^27^). NMDA receptors are ionotropic glutamate receptors subject to voltage-dependent block by Mg^2+^ ions^28^ and are noncompetitively inhibited by Zn^2+^ ions.^29^ Although the functions of NMDA receptors in the retina are not entirely clear, a defect in either Mg^2+^ or Zn^2+^ transport is likely to lead to abnormal functioning of these receptors and their parent cells. GABA receptors modulate neurotransmitter release in the retina^30^ and are also inhibited by Zn^2+^ ions.^29^ As such, failure of Zn^2+^ transport could lead to excitotoxic conditions in the retina. Future work will be required to confirm whether CNNM4 shares the magnesium-transport function of CNNM2 or whether it is involved in the transport of other metal ions.

Like CNNM2, CNNM4 is predicted to have four transmembrane helices and contains a cyclic nucleotide-binding domain, two cystathionine-beta-synthase (CBS) domains, and a DUF21 domain of unknown function. CBS domains appear to have a role in sensing the energy status of cells by binding to ATP and are present in proteins such as CLC chloride channels and IMPDH1, mutations in which are a known cause of retinal degeneration.^31^ Furthermore, cyclic nucleotide-binding domains are present in the cyclic nucleotide-gated channels of photoreceptors. These channels are involved in ion transport and maintaining the photoreceptor dark current, and they are closed by reduction in cGMP levels upon phototransduction. Several of the protein subunits making up these channels are implicated in retinal disease.^32^, ^33^, ^34^, ^35^ On the basis of homology to CNNM2, CNNM4 is likely to behave as a transporter rather than a channel, and its localization in the retina as determined by immunofluorescence suggests that its role is quite different from that of cyclic nucleotide-gated channels. However, it will be interesting to determine whether CNNM4 has an ion-transport function that can be regulated by cyclic nucleotides in a similar manner.

The finding that mutations in *CNNM4* have clinical consequences that are limited to retinal function and biomineralization is surprising given the wide tissue distribution of the encoded protein,^17^, ^36^ but it is far from unprecedented. To our knowledge, this is only the second gene to be identified as a cause of AI with a purely genetic approach. Although immunolocalization data support a role for CNNM4 in retinal function and biomineralization, further work will be required to uncover its function in these tissues. This gene discovery not only suggests the intriguing possibility of a direct link between tooth biomineralization and retinal function, but it may also give a novel insight into the role of metal transport in these processes.

## Web Resources

The URLs for data presented herein are as follows:

Online Mendelian Inheritance in Man (OMIM), http://www.ncbi.nlm.nih.gov/Omim/
RetNet, the Retinal Information Network, http://www.sph.uth.tmc.edu/RetNet/

## Supplemental Data

Document S1. Four Tables

Document S2. One Table
