# Supplementary material for: Mutations in CNNM4 Cause Jalili Syndrome, Consisting of Autosomal-Recessive Cone-Rod Dystrophy and Amelogenesis Imperfecta
Source: Am J Hum Genet. 2009 Feb 13;84(2):266–73. doi: 10.1016/j.ajhg.2009.01.009 (PMC2668026; doi:10.1016/j.ajhg.2009.01.009)
Supplement: Document S1. Four Tables [file mmc1.pdf]

AJHG, Volume *84*

Supplemental Data

Mutations in *CNNM4* cause Jalili syndrome, consisting of autosomal recessive cone-rod dystrophy and amelogenesis imperfecta

David A Parry*, Alan J Mighell*, Walid El-Sayed, Roger C Shore, Ismail K Jalili, Hélène Dollfus, Agnes Bloch-Zupan, Roman Carlos, Ian M Carr, Louise M Downey, Katharine M Blain, David C Mansfield, Mehdi Shahrabi, Mansour Heidari, Parissa Aref, Mohsen Abbasi, Michel Michaelides, Anthony T Moore, Jennifer Kirkham, Chris F Inglehearn.

---

* These authors contributed equally to this work.

Table S1. Candidate genes screened for mutations.

2q11 genes screened for mutations in Jalili Syndrome families. Four novel SNPs (2 synonymous and 2 intronic changes) discovered during screening are shown with respective Submitter SNP ID (SS) numbers. The pathogenic changes in the Jalili syndrome families are also shown. Initial screening was performed on genomic DNA from affected members of the Gaza A, Gaza B and Guatemala families.

Gene	Entrez Gene	OMIM	Novel SNPs	Pathogenic change 1	Pathogenic change 2
ACTR1B	10120	*605144	-	-	-
CNGA3	1261	*600053	-	-	-
CNNM3	26505	*607804	-	-	-
CNNM4	26504	*607805	c.1350C>T (p.=) [Unaffected control] (SS115492347)	c.599C>A; Ser200Tyr [Gaza A]	c.599C>A; Ser200Tyr [Gaza A]
				c.1312dupC; Leu438ProfsX9 [Kosovo]	c.1312dupC; Leu438ProfsX9 [Kosovo]
				c.1813 C>T; Arg605X [Gaza B]	c.1813 C>T; Arg605X [Gaza B]
				c.2149C>T; Gln717X [Guatemala]	c.62_145del; Leu21HisfsX185 [Guatemala]
				c.586T>C; Ser196Pro [Turkey]	c.586T>C; Ser196Pro [Turkey]
				c.1-?_1403+?del [Iran]	c.1-?_1403+?del [Iran]
				c.971T>C; Leu324Pro [Scotland]	c.1690C>T; Gln564X [Scotland]
INPP4A	3631	*600916	-	-	-
KCNIP3	30818	*604662	-	-	-
MGAT4A	11320	*604623	-	-	-
PROM2	150696	-	c.831G>A (p.=) [Gaza B] (SS115492348)	-	-
			c.975+21C>A (p.=) [Guatemala] (SS115492350)		
			c.1644-25G>A (p.=) [Gaza B] (SS115492349)		
SEMA4C	54910	604462	-	-	-
UNC50	25972	-	-	-	-
ZNF2	*7549*	**194500*	-	-	-

Table S2. Gaza B family microsatellite genotypes.

Microsatellite makers genotyped in the Gaza B family. Analysis indicated linkage to 2q11 with a large region of homozygosity overlapping with the published locus on 2q11 in affected individuals. The shared affected haplotype is shown in red.

Marker ID	Chromosome 2 Physical Position (bp)	Alleles							
		IV:1	IV:2	(Aff) V:1	V:2	(Aff) V:3	(Aff) V:4	V:5	V:6
D2S2216	88190756	136 144	142 144	144 144	142 144	144 144	136 144	136 144	136 142
D2S2181	88457696	171 187	171 179	171 171	171 179	171 171	171 187	171 187	179 187
	Centromere								
D2S2154	94912173	275 275	275 275	275 275	275 275	275 275	275 275	275 275	275 275
D2S2159	95413502	175 179	175 177	175 175	175 177	175 175	175 175	175 179	177 179
D2S113	96641271	209 223	209 215	209 209	209 215	209 209	209 209	209 223	215 223
D2S1258	97366093	181 181	181 185	181 181	181 185	181 181	181 181	181 181	181 185
D2S2222	97665780	219 221	219 221	221 221	219 221	221 221	221 221	219 221	219 219
D2S2175	98151116	125 129	123 125	125 125	123 125	125 125	125 125	125 129	123 129
D2S2311	98383050	150 154	150 154	154 154	150 154	154 154	154 154	150 154	150 150
D2S2187	98471639	142 152	142 154	142 142	142 154	142 142	142 142	142 152	152 154
D2S1309	99057069	74 88	74 84	74 74	74 84	74 74	74 74	74 88	84 88
D2S2209	100608611	190 190	184 190	190 190	184 190	190 190	190 190	190 190	184 190
D2S2264	101785989	245 245	239 245	245 245	239 245	245 245	245 245	245 245	239 245
D2S2972	101938400	228 228	228 228	228 228	228 228	228 228	228 228	228 228	228 228
D2S373	102507335	222 230	222 232	222 222	222 232	222 222	222 222	222 230	230 232
D2S2356	102872869	128 128	122 128	128 128	122 128	128 128	128 128	128 128	122 128

Table S3. Guatemala family microsatellite genotypes.

Microsatellite markers genotyped in the Guatemalan family. Analysis indicated linkage to 2q11 with the shared affected haplotypes shown in red. There was no family history of consanguinity and genotyping confirmed compound heterozygosity at the disease locus.

Marker ID	Chromosome 2 Physical Location (bp)	Alleles													
		I:1	I:2	II:1	II:2	(Aff) II:3	(Aff) II:4	(Aff) II:5	(Aff) II:6	II:7	II:8	II:9	II:10	II:11	III:1
D2S113	96641271	222 222	206 212	? ?	? ?	212 222	212 222	212 222	212 222	206 222	? ?	212 222	206 221	? ?	220 206
D2S1258	97366093	177 181	181 173	? ?	? ?	173 181	173 181	173 181	173 181	181 177	? ?	181 177	181 177	? ?	177 181
D2S2222	97665780	222 218	224 224	? ?	? ?	224 218	224 218	224 218	224 218	224 218	? ?	224 222	224 222	? ?	222 224
D2S2175	98151116	125 125	121 121	? ?	? ?	121 125	121 125	121 125	121 125	121 125	? ?	121 125	121 125	? ?	123 121
D2S1309	99057069	89 89	83 81	? ?	? ?	81 89	81 89	81 89	81 89	83 89	? ?	81 89	83 89	? ?	83 83
D2S2209	100608611	192 196	196 186	? ?	? ?	186 196	186 196	186 196	186 196	196 196	? ?	186 192	196 192	? ?	195 195
D2S2264	101785989	229 249	249 239	? ?	? ?	239 249	239 249	239 249	239 249	249 249	? ?	239 229	249 229	? ?	247 249
D2S2972	101938400	229 237	237 233	? ?	? ?	233 237	233 237	233 237	233 237	237 237	? ?	233 229	237 229	? ?	229 237
D2S373	102507335	222 233	231 233	? ?	? ?	233 233	233 233	233 233	233 233	231 233	? ?	233 222	231 222	? ?	222 231
D2S2356	102872869	126 126	114 126	? ?	? ?	114 126	126 126	126 126	126 126	114 126	? ?	126 126	126 126	? ?	120 126

Table S4. Iran family microsatellite genotypes.

Microsatellite markers genotyped in the Iranian family. Haplotype analysis provided support for the existence of a homozygous linked region in affected individuals within the disease locus on 2q11, shown in red.

Marker ID	Chromosome 2 Physical Location (bp)	Alleles								
		III:1	III:2	(Aff) IV:1	(Aff) IV:2	(Aff) IV:3	IV:4	IV:5	IV:6	(Aff) V:1
D2S113	96641271	214 222	214 218	214 214	214 214	214 214	214 222	212 214	? ?	214 214
D2S2222	97665780	? ?	220 222	222 222	222 222	222 222	222 226	222 224	? ?	222 222
D2S2175	98151116	123 123	121 123	123 123	123 123	123 123	123 123	123 123	? ?	123 123
D2S2264	101785989	245 247	245 247	245 245	245 245	245 245	245 247	245 245	? ?	245 245
D2S2972	101938400	229 229	229 237	229 229	229 229	229 229	229 229	229 237	? ?	229 229
D2S373	102507335	232 232	232 232	232 232	232 232	232 232	232 232	232 232	? ?	232 232
