# Supplementary material for: Mutations in CNNM4 Cause Jalili Syndrome, Consisting of Autosomal-Recessive Cone-Rod Dystrophy and Amelogenesis Imperfecta
Source: Am J Hum Genet. 2009 Feb 13;84(2):266–73. doi: 10.1016/j.ajhg.2009.01.009 (PMC2668026; doi:10.1016/j.ajhg.2009.01.009)
Supplement: Document S2. One Table [file mmc2.pdf]

Table S5. Genotyping by Affymetrix Genome-Wide Human SNP Array 6.0.

Shown are SNP genotypes in the vicinity of the linked region on chromosome 2. The refined region of homozygosity between RS2628473 and RS1901284 is highlighted in blue. SNPs with one or more "No call" have been deleted for clarity. No significant region of homozygosity was discovered in the Guatemalan or Scottish families which were subsequently found to be compound heterozygotes for mutations in CNNM4. The Iranian family was not subject to SNP array analysis.

SNP ID	Position	Gaza A Affected (VIII:10)	Gaza B Affected (V:4)	Turkey Affected (II:2)	Kosovo Affected (II:1)	Kosovo Affected (II:2)
RS13383174	83,397,510	AA	AB	AA	AA	AA
RS6745924	83,404,580	BB	BB	AA	AA	AA
RS9631052	83,420,160	AA	AB	AA	AA	AA
RS7563569	83,443,860	AA	AB	BB	BB	BB
RS1429398	83,458,300	AA	AA	AA	AA	AA
RS1121554	83,461,580	AA	AA	AA	AA	AA
RS1035979	83,467,540	BB	BB	BB	BB	AB
RS17380093	83,490,570	BB	AB	BB	AB	AB
RS11887003	83,510,330	AA	AB	AA	AA	AA
RS17023490	83,515,780	AA	AA	AA	AA	AA
RS7578567	83,521,580	AA	AA	AA	AA	AA
RS17023511	83,524,740	AA	AB	AA	AA	AA
RS13428591	83,525,880	AA	AB	AA	AB	AB
RS17023566	83,535,990	AA	AA	AA	AA	AA
RS13384567	83,536,610	AA	AB	AA	AA	AA
RS13384589	83,536,660	BB	AB	BB	BB	BB
RS6547499	83,540,670	AA	AB	AA	AA	AA
RS17023589	83,543,010	AA	AA	AA	AA	AA
RS967016	83,548,440	AB	BB	AA	AB	AB
RS9309601	83,548,580	AB	AB	BB	BB	BB
RS17023626	83,557,980	BB	AB	BB	BB	BB
RS13384894	83,564,820	BB	AB	BB	BB	BB
RS13392429	83,567,150	AA	AB	AA	AA	AA
RS12619848	83,568,060	BB	AB	BB	AB	AB
RS9631074	83,594,910	AA	AB	AA	AA	AA
RS6706752	83,602,990	BB	AB	BB	AB	AB
RS1366827	83,606,800	AA	BB	AA	AB	AB
RS1366828	83,606,970	AA	AB	AA	AB	AB
RS13015327	83,615,580	AA	AB	AA	AB	AB
RS1011477	83,626,310	AA	AB	AA	AB	AB
RS17315158	83,632,110	AA	AB	AA	AA	AA
RS6740151	83,634,350	AB	AA	AB	AB	AB
RS17023704	83,655,490	BB	BB	BB	BB	BB
RS1486837	83,659,770	BB	AB	BB	BB	BB
RS17023713	83,660,860	BB	BB	BB	BB	BB
RS6722452	83,660,880	BB	BB	BB	BB	BB
RS1386306	83,662,820	BB	AA	BB	AA	AB
RS6747341	83,676,540	AA	AA	AA	AA	AA

RS1906128	83,696,210	AA	BB	AA	AB	AB
RS10520375	83,696,280	AA	AA	AA	AA	AA
RS10173571	83,711,460	AB	AA	AA	AA	AA
RS11682212	83,722,620	BB	AA	BB	AB	AB
RS11894402	83,729,630	AA	AA	AA	AA	AA
RS7590611	83,744,920	AA	BB	AA	AA	AA
RS17023794	83,760,020	AA	AB	AA	AA	AA
RS1872734	83,760,980	BB	AB	BB	BB	BB
RS1868657	83,765,660	BB	AB	BB	BB	BB
RS1901396	83,771,980	AA	BB	AA	AA	AA
RS896476	83,777,300	AA	BB	AA	AA	AA
RS10496299	83,783,490	AA	AB	AA	AA	AA
RS12052366	83,783,700	AA	AB	AA	AA	AB
RS10195787	83,790,250	AA	AB	AA	AA	AA
RS13020687	83,797,800	AA	BB	AA	AA	AA
RS2859990	83,800,130	AA	AB	AA	AA	AA
RS2198006	83,800,320	AA	AB	AA	AB	AB
RS11897350	83,802,460	AA	AB	AA	AB	AB
RS12151569	83,814,860	BB	AB	BB	AB	AB
RS1450263	83,816,510	AB	BB	BB	BB	AB
RS1486834	83,817,170	AA	AB	AA	AB	AB
RS4832321	83,822,640	AA	AB	AA	AB	AB
RS7576097	83,825,590	AA	AB	AA	AB	AB
RS17024065	83,832,620	BB	BB	BB	BB	BB
RS2068677	83,835,740	BB	AB	BB	AB	AB
RS17024079	83,835,860	AA	AB	AA	AA	AA
RS1115900	83,841,980	BB	BB	BB	BB	BB
RS2122456	83,845,110	AA	AA	AA	AA	AA
RS1450253	83,865,920	BB	BB	BB	BB	BB
RS3100102	83,867,090	AA	AA	AA	AA	AA
RS4832325	83,883,090	BB	AB	BB	AB	AB
RS10169744	83,902,100	AA	AB	AA	AB	AB
RS4832333	83,908,430	BB	AB	BB	AB	AB
RS10178418	83,909,740	BB	AB	BB	AB	AB
RS10202321	83,909,830	BB	AB	BB	AB	AB
RS12618752	83,917,490	AA	AA	AA	AA	AA
RS4832061	83,919,520	BB	BB	BB	BB	BB
RS13423886	83,927,820	AA	AA	AA	AA	AA
RS11896453	83,928,450	BB	BB	BB	BB	BB
RS6727508	83,931,670	BB	BB	BB	BB	BB
RS13409188	83,941,220	AA	AA	AA	AA	AA
RS4832348	83,941,260	AA	AA	AA	AA	AA
RS12714112	83,946,270	AA	AA	AA	AA	AB
RS4443013	83,950,990	BB	BB	BB	BB	BB
RS10865470	83,959,470	AA	AA	AA	AA	AA
RS10170098	83,960,090	AA	AA	AA	AA	AA
RS4550682	83,962,620	AA	AA	AA	AA	AA
RS7566318	83,969,500	AA	AA	AA	AA	AA
RS4528772	83,991,380	BB	BB	BB	BB	BB
RS10865472	84,006,230	AA	AA	AA	AA	AB
RS4832352	84,008,570	AA	AA	AA	AA	AA

RS17024248	84,009,590	BB	BB	BB	BB	BB
RS1374731	84,019,860	AA	AA	AA	AA	AA
RS12328713	84,022,390	AA	AA	AA	AA	AA
RS12616175	84,036,170	AA	AA	AA	AA	AA
RS2885856	84,049,500	AA	AA	AA	AA	AA
RS2885857	84,049,580	BB	BB	BB	BB	BB
RS725729	84,052,380	BB	BB	BB	BB	BB
RS10189816	84,054,220	BB	BB	BB	BB	BB
RS10190107	84,054,570	BB	BB	BB	BB	BB
RS12714117	84,054,630	BB	BB	BB	BB	BB
RS12162398	84,056,640	AA	AA	AA	AA	AA
RS763866	84,071,800	AA	AA	AA	AA	AB
RS13401483	84,072,680	BB	BB	BB	BB	BB
RS1348954	84,073,490	AA	AA	AA	AA	AA
RS17024345	84,074,460	AA	AA	AA	AA	AA
RS4832366	84,080,590	AA	AA	AA	AA	AA
RS7576982	84,084,970	AA	AA	AA	AA	AA
RS11126939	84,103,480	BB	BB	BB	BB	BB
RS11126941	84,106,920	BB	BB	BB	BB	BB
RS12474795	84,112,840	BB	BB	BB	BB	BB
RS1348955	84,117,280	BB	BB	BB	BB	BB
RS17024420	84,120,550	AA	AA	AA	AA	AA
RS17024458	84,134,140	BB	BB	BB	BB	BB
RS13409074	84,135,250	BB	BB	BB	BB	BB
RS2100129	84,137,560	AA	AA	AA	AA	AA
RS4832371	84,138,210	AA	AA	AA	AA	AA
RS17024465	84,138,780	BB	BB	BB	BB	BB
RS17388373	84,138,880	AA	BB	AA	BB	BB
RS13431095	84,141,110	AA	AA	AA	AA	AA
RS1584308	84,144,100	BB	BB	BB	BB	BB
RS17024540	84,168,930	AA	AA	AA	AA	AA
RS17024543	84,169,040	BB	BB	BB	BB	BB
RS1597039	84,177,540	BB	AB	BB	AB	AB
RS6705963	84,180,580	AA	AB	AA	AB	AB
RS11884424	84,196,900	BB	AB	BB	AB	AB
RS1526700	84,199,190	AA	AB	AA	AB	AB
RS1918095	84,200,750	AA	AB	AA	AB	AB
RS11674417	84,202,140	BB	BB	BB	BB	BB
RS7579223	84,215,780	BB	AB	BB	AB	AB
RS7579456	84,215,970	AA	AB	AA	AB	AB
RS4263122	84,216,110	BB	BB	BB	BB	BB
RS1568997	84,222,110	AA	AB	AA	AB	AB
RS4453706	84,223,690	AA	AB	AA	AB	AB
RS2364236	84,225,460	AA	AA	AA	AB	AB
RS11884557	84,227,140	BB	BB	BB	BB	BB
RS1358194	84,234,960	AA	AB	AA	AA	AB
RS13385328	84,239,140	AA	AA	AA	AA	AA
RS17024618	84,260,580	AA	AB	AA	AA	AA
RS17024623	84,263,750	AA	AA	AA	AA	AA
RS10496308	84,273,240	BB	AA	BB	AB	AB
RS11126951	84,273,500	AA	AB	AA	AB	AB

RS6736384	84,280,860	BB	AB	BB	AB	AB
RS10180739	84,282,330	BB	AB	BB	AB	AB
RS2886029	84,285,010	BB	AB	BB	AB	AB
RS10865473	84,287,150	BB	AB	BB	AB	AB
RS11126953	84,287,260	BB	AB	BB	AB	AB
RS11126954	84,287,410	BB	AB	BB	AB	AB
RS6727246	84,290,990	AA	AB	AA	AB	AB
RS2364258	84,295,340	AA	AB	AA	AB	AB
RS12714122	84,297,380	AA	AB	AA	AB	AB
RS6750854	84,302,450	AA	AB	AA	AB	AB
RS2364265	84,313,510	BB	AB	BB	AB	AB
RS2364266	84,313,540	BB	AB	BB	AB	AB
RS4020792	84,314,380	AA	AB	AA	AB	AB
RS2364267	84,317,740	BB	AB	BB	AB	AB
RS11899552	84,323,540	AA	AA	AA	AA	AA
RS7602393	84,324,020	BB	AB	BB	AB	AB
RS744513	84,324,620	AA	AB	AA	AB	AB
RS12466935	84,326,410	BB	AB	BB	AB	AB
RS11126958	84,330,450	AA	AB	AA	AB	AB
RS6547544	84,331,330	BB	AB	BB	AB	AB
RS6761743	84,331,460	BB	AB	BB	AB	AB
RS6547547	84,340,240	BB	AB	BB	AB	AB
RS17024780	84,340,340	BB	BB	BB	BB	BB
RS882501	84,358,060	AA	AA	AA	AB	AB
RS731331	84,362,530	AA	AA	AA	AA	AA
RS731330	84,362,710	AA	AB	AA	AB	AB
RS1869666	84,364,540	BB	BB	BB	AB	AB
RS1031532	84,373,180	AA	AB	AA	AB	AB
RS2168224	84,378,180	AA	AA	AA	AB	AB
RS2124177	84,378,450	BB	AB	BB	AB	AB
RS11686691	84,382,260	BB	AB	BB	AB	AB
RS2219161	84,387,840	BB	BB	BB	AB	AB
RS2061080	84,388,490	AA	AB	AA	AB	AB
RS7572995	84,390,470	BB	BB	BB	BB	BB
RS7572915	84,390,500	BB	BB	BB	BB	BB
RS17024868	84,390,590	BB	BB	BB	AB	AB
RS4832071	84,402,200	AA	AA	AA	AB	AB
RS7601141	84,402,820	AA	AA	AA	AB	AB
RS1454998	84,419,040	BB	AA	BB	AB	AB
RS10211465	84,419,700	AA	AA	AA	AB	AB
RS1840406	84,429,910	BB	BB	BB	AB	AB
RS1947463	84,433,010	AA	AA	AA	AB	AB
RS11890635	84,440,550	AA	AA	AA	AA	AA
RS1455004	84,442,050	AA	AA	AA	AB	AB
RS10496309	84,468,980	BB	BB	BB	BB	BB
RS2085192	84,470,010	BB	BB	BB	BB	BB
RS1947465	84,471,620	BB	BB	BB	AB	AB
RS12613583	84,474,660	AA	AA	AA	AA	AA
RS11686391	84,476,670	BB	AB	BB	BB	BB
RS11126962	84,491,620	AA	AA	AA	AA	AA
RS17025000	84,493,750	AA	AA	AA	AA	AA

RS13383562	84,493,810	AA	AA	AA	AA	AA
RS17757342	84,497,120	AA	BB	BB	BB	BB
RS2832	84,504,220	AA	AA	AA	AB	AB
RS4831973	84,510,950	AA	AA	AA	AB	AB
RS12618709	84,515,870	BB	BB	BB	BB	BB
RS17757829	84,520,140	BB	BB	AB	BB	BB
RS1599306	84,525,990	AA	AA	AA	AB	AB
RS6719880	84,526,860	AA	AA	AA	AB	AB
RS13000280	84,550,500	BB	BB	BB	AB	AB
RS13006689	84,550,950	BB	AB	BB	BB	BB
RS7590348	84,553,270	BB	BB	BB	BB	BB
RS12623199	84,554,010	BB	BB	BB	BB	BB
RS4832087	84,578,180	AA	AA	AA	AB	AB
RS4831979	84,578,890	AA	AB	AA	AB	AB
RS12373700	84,633,000	AA	AA	AA	AB	AB
RS13034783	84,636,260	AA	AB	AA	AA	AA
RS7559623	84,639,690	AA	AA	AA	AB	AB
RS17759625	84,669,190	AA	BB	AA	BB	BB
RS1460703	84,669,260	AB	BB	BB	BB	BB
RS1006244	84,669,530	AA	AB	AA	AA	AA
RS7559641	84,669,830	AA	AA	AA	AA	AA
RS4020077	84,681,510	AA	AB	AA	AB	AB
RS1460710	84,684,420	AA	AB	AA	AB	AB
RS1460709	84,684,780	BB	AB	BB	AB	AB
RS7570369	84,685,280	BB	AB	BB	AB	AB
RS12997105	84,687,140	BB	AB	BB	AB	AB
RS7587820	84,695,130	AA	AB	AA	AB	AB
RS11889456	84,700,190	BB	AB	BB	AB	AB
RS11126970	84,705,270	AA	AB	AA	AB	AB
RS11676670	84,705,520	AA	AB	AA	AB	AB
RS6726617	84,713,400	BB	AB	BB	AB	AB
RS4313996	84,716,350	AA	AB	AA	AB	AB
RS7572042	84,720,760	AA	AB	AA	AB	AB
RS7593359	84,728,470	AA	AB	AA	AB	AB
RS11895477	84,752,760	AA	AB	AA	AA	AA
RS1918697	84,754,380	AA	AB	BB	AB	AB
RS1918695	84,755,720	AA	BB	BB	AB	AB
RS17759986	84,756,190	BB	BB	BB	BB	BB
RS17025450	84,760,300	BB	BB	BB	BB	BB
RS6749125	84,760,880	AA	AB	BB	AB	AB
RS17025484	84,769,910	BB	BB	BB	BB	BB
RS1192304	84,770,400	AA	AA	AA	AB	AB
RS1192296	84,773,550	BB	BB	BB	AB	AB
RS1192285	84,779,990	BB	BB	BB	AB	AB
RS1192269	84,786,340	AA	AA	AA	AB	AB
RS1192266	84,789,710	BB	BB	BB	AB	AB
RS1918693	84,792,500	BB	AB	AA	BB	BB
RS17025508	84,798,620	AA	AA	AA	AA	AA
RS10199591	84,799,580	AA	AB	AB	AA	AA
RS1192333	84,805,190	AA	AB	BB	AB	AB
RS1192324	84,815,250	BB	BB	BB	AB	AB

RS10180799	84,826,420	BB	AB	AA	BB	BB
RS890615	84,831,890	BB	AB	AA	AB	AB
RS17025543	84,846,170	BB	AB	AA	BB	BB
RS7606803	84,869,020	BB	BB	BB	BB	BB
RS1192395	84,878,240	BB	AB	AA	BB	BB
RS1192367	84,887,340	BB	AB	AB	BB	BB
RS7573165	84,888,240	BB	BB	BB	BB	BB
RS1192366	84,888,570	AA	AB	BB	AB	AB
RS11686936	84,889,450	AA	AA	AA	AB	AB
RS7584208	84,899,980	AA	AA	AA	AA	AA
RS7584703	84,900,430	AA	AA	AA	AA	AA
RS17025617	84,901,680	AA	AA	AA	AA	AA
RS1627220	84,906,970	AA	AB	BB	AA	AA
RS1192277	84,909,340	AA	AB	BB	AA	AA
RS7579460	84,911,570	AA	AA	AA	AB	AB
RS12472846	84,919,140	AA	AB	BB	AB	AB
RS10514646	84,922,140	AA	AA	AA	AA	AA
RS10514645	84,931,290	AA	AA	BB	AB	AB
RS17025647	84,931,950	BB	BB	BB	BB	BB
RS6743110	84,932,540	AA	AA	BB	BB	BB
RS735797	84,942,930	AA	AA	AA	AB	AB
RS11693789	84,955,250	AA	AA	AA	AB	AB
RS1478782	84,957,660	AA	AA	AA	AB	AB
RS4375877	84,975,820	AA	AA	AA	AA	AA
RS4377349	84,975,920	AA	AA	AA	AA	AA
RS6547580	84,980,700	BB	AB	BB	BB	BB
RS6547581	84,980,780	BB	AB	BB	BB	BB
RS3884258	85,038,670	BB	BB	BB	BB	BB
RS3883876	85,040,670	AA	AB	BB	BB	BB
RS6714113	85,042,660	AA	AB	BB	BB	BB
RS17038088	85,044,100	BB	BB	BB	BB	BB
RS17038084	85,045,790	BB	AB	BB	BB	BB
RS17025771	85,067,630	BB	BB	BB	BB	BB
RS17710619	85,093,820	AA	AA	AA	AB	AB
RS17498115	85,102,810	AA	AA	AA	AA	AA
RS17025820	85,123,950	BB	BB	BB	BB	BB
RS17025827	85,128,180	BB	BB	BB	BB	BB
RS12616403	85,133,550	AA	AA	AA	AA	AA
RS1882271	85,142,080	BB	BB	BB	BB	BB
RS11690856	85,145,340	BB	AB	AA	AA	AA
RS10191268	85,145,700	BB	AB	BB	BB	BB
RS12623715	85,162,020	AA	AA	AB	AA	AB
RS2583569	85,175,260	AA	AA	BB	AB	AB
RS7563120	85,177,730	BB	BB	BB	BB	BB
RS2583556	85,185,200	AA	AA	AA	AB	AB
RS11693890	85,185,930	BB	BB	AA	AB	AB
RS11682512	85,185,950	AA	AA	BB	AB	AB
RS4832135	85,187,490	AA	AA	AA	AB	AB
RS11884056	85,193,350	AA	AB	AA	AA	AA
RS10496315	85,193,570	AA	AA	BB	AA	AA
RS17025915	85,195,340	BB	BB	BB	BB	BB

RS17025918	85,196,200	BB	BB	AA	BB	BB
RS6728374	85,212,060	BB	BB	AA	AB	AB
RS1835812	85,219,010	BB	BB	BB	AB	AB
RS10520405	85,228,700	AA	AA	AA	AA	AA
RS17025940	85,228,790	AA	AA	AA	AA	AA
RS2568219	85,229,090	BB	AB	AA	BB	BB
RS6754217	85,229,180	AA	AA	AA	AA	AA
RS17025951	85,234,580	AA	AA	AA	AA	AA
RS17762406	85,234,930	BB	AB	BB	BB	BB
RS13402724	85,243,950	AA	AA	AA	AA	AA
RS17025984	85,247,780	BB	BB	BB	BB	BB
RS11691849	85,248,120	AB	AB	AA	AA	AA
RS11682047	85,248,620	AA	AB	BB	AB	AB
RS4832145	85,254,100	BB	BB	BB	BB	BB
RS17025989	85,256,000	AA	AA	AA	AA	AA
RS2568203	85,264,450	AA	AB	BB	AA	AA
RS4374376	85,274,220	AA	AA	AA	AA	AA
RS10192927	85,282,370	BB	BB	BB	BB	BB
RS12619496	85,294,910	BB	BB	BB	BB	BB
RS13396934	85,297,330	BB	BB	BB	BB	BB
RS6709476	85,297,430	AA	AB	BB	BB	BB
RS6715431	85,300,540	AA	AA	AA	AA	AA
RS11892343	85,304,330	BB	AB	BB	BB	BB
RS10205484	85,321,510	AA	AB	BB	AA	AA
RS7598881	85,323,500	BB	BB	BB	BB	BB
RS11675205	85,323,790	AB	BB	BB	BB	BB
RS17026102	85,329,220	BB	AB	BB	BB	BB
RS17763853	85,334,220	BB	AB	AA	AA	AA
RS7581133	85,334,330	AA	AB	AA	BB	BB
RS17713036	85,334,910	AA	AA	BB	AA	AA
RS7584666	85,341,960	BB	AB	BB	AA	AA
RS11689667	85,344,880	BB	AB	AA	AA	AA
RS6547607	85,344,930	AA	AB	AA	BB	BB
RS10201489	85,354,920	BB	BB	BB	AB	AB
RS12714137	85,355,780	BB	BB	BB	AB	AB
RS12714138	85,355,930	AA	BB	BB	AB	AB
RS6747629	85,359,070	BB	BB	BB	AB	AB
RS6733190	85,359,430	BB	AA	AA	AA	AA
RS6748258	85,359,490	AA	AA	AA	AB	AB
RS10165984	85,360,100	BB	BB	BB	AB	AB
RS7598047	85,363,530	AA	BB	AA	AA	AA
RS7600828	85,363,810	AA	BB	BB	BB	BB
RS7574999	85,364,480	BB	BB	BB	AA	AA
RS17026157	85,365,160	BB	BB	BB	BB	BB
RS17713693	85,365,580	BB	BB	AB	BB	BB
RS6719271	85,368,220	BB	BB	BB	BB	BB
RS6706073	85,371,780	BB	BB	BB	BB	BB
RS4832156	85,376,780	AA	AB	BB	AA	AA
RS1053561	85,399,560	BB	AB	AA	AB	AB
RS6547611	85,402,150	BB	AB	AA	AB	AB
RS6747513	85,419,140	AA	AB	BB	AB	AB

RS17038362	85,421,920	BB	BB	BB	BB	BB
RS1969260	85,422,320	BB	AB	AA	AB	AB
RS999939	85,422,810	BB	BB	BB	BB	BB
RS15413	85,422,890	AA	AA	AA	AA	AA
RS6547615	85,427,780	BB	AB	AA	AB	AB
RS7340340	85,447,620	BB	AB	AA	BB	AB
RS12104980	85,449,540	AA	AA	AA	AA	AA
RS6731223	85,458,820	BB	BB	BB	BB	BB
RS11680448	85,464,550	AA	AA	AA	AA	AA
RS1135604	85,468,520	AA	AB	AA	AA	AA
RS17026285	85,470,270	BB	BB	BB	BB	BB
RS11682055	85,491,830	AA	AB	BB	AB	AB
RS1877954	85,507,810	AA	BB	BB	BB	BB
RS17026339	85,525,840	BB	BB	BB	BB	BB
RS908307	85,527,500	AA	AA	AA	AA	AA
RS10496317	85,536,880	AA	BB	AA	AB	AB
RS2121395	85,537,080	BB	AA	BB	AB	AB
RS17735501	85,538,720	BB	BB	BB	BB	BB
RS17026356	85,538,980	AA	BB	AA	AB	AB
RS7571301	85,539,250	BB	AA	BB	BB	BB
RS12616455	85,550,060	AA	AB	AA	AA	AA
RS960066	85,554,430	AA	AB	AA	AB	AB
RS2166529	85,595,690	AA	AB	AA	AA	AA
RS2886722	85,595,810	AA	AB	AA	AA	AA
RS7579665	85,596,130	BB	AB	BB	AB	AB
RS10496316	85,600,880	BB	BB	BB	AB	AB
RS17026396	85,612,640	AA	AB	AA	AA	AA
RS2028900	85,621,250	AA	AB	AA	AA	AA
RS11675092	85,621,380	AA	AA	AA	AA	AA
RS2043675	85,623,490	AA	AB	AA	AB	AB
RS6723678	85,629,660	AA	AA	AA	AA	AA
RS699664	85,634,050	AA	AB	AA	AB	AB
RS13429968	85,651,180	BB	BB	BB	AB	AB
RS13426038	85,656,910	AA	AA	AA	AB	AB
RS6757263	85,657,060	BB	AB	AA	BB	BB
RS1010	85,662,500	AA	AB	BB	AA	AA
RS7593969	85,663,180	AA	AB	BB	AB	AB
RS1561198	85,663,500	BB	AB	AA	BB	BB
RS1254900	85,669,850	AA	AA	AA	AA	AA
RS1374370	85,671,780	BB	AB	AA	BB	BB
RS719023	85,672,000	AA	AA	BB	AA	AA
RS6742887	85,674,710	AA	AA	AA	AA	AA
RS2232740	85,676,330	AA	AA	AA	AA	AA
RS6547629	85,713,740	BB	BB	AA	BB	BB
RS7579847	85,714,140	AA	AB	AA	AA	AA
RS3821020	85,723,430	BB	BB	BB	BB	BB
RS13386893	85,730,590	AA	AA	AA	AA	AA
RS7316	85,739,520	AA	AA	AA	AA	AA
RS3024811	85,743,660	AA	AB	AA	AA	AA
RS2118177	85,743,810	BB	BB	AA	BB	BB
RS1130866	85,747,250	AA	AA	BB	AB	AB

RS6547631	85,762,540	AA	AA	BB	AB	AB
RS10180445	85,779,730	AA	AB	BB	AB	AB
RS10210321	85,781,640	BB	AB	AA	AB	AB
RS6759087	85,802,300	AA	AB	BB	AB	AB
RS11126997	85,802,900	AA	AA	BB	AB	AB
RS9309624	85,813,620	BB	BB	BB	BB	BB
RS2176625	85,815,360	AA	AA	AA	AA	AA
RS7425090	85,815,660	AA	AA	AA	AA	AA
RS6727520	85,818,990	AA	AA	AA	AA	AA
RS1437741	85,822,090	AA	AB	BB	AB	AB
RS13430978	85,824,220	BB	AB	AA	AB	AB
RS13431188	85,824,460	BB	BB	BB	BB	BB
RS17026608	85,826,140	AA	AA	AA	AA	AA
RS1356623	85,826,430	BB	AB	BB	BB	BB
RS1356621	85,826,620	AB	AA	AA	AA	AA
RS1465822	85,829,900	AA	AA	AA	AA	AA
RS11126998	85,832,300	BB	BB	BB	BB	BB
RS4832198	85,849,160	BB	AB	AA	AB	AB
RS6746541	85,855,140	BB	AB	AA	AB	AB
RS934775	85,858,410	AA	AB	BB	AA	AA
RS934776	85,858,740	BB	BB	BB	BB	BB
RS2139198	85,861,180	AA	AA	AA	AA	AA
RS13429794	85,863,870	AA	AA	AA	AA	AA
RS13386681	85,866,540	BB	BB	BB	BB	BB
RS13402783	85,868,630	AA	AA	AA	AA	AA
RS13428557	85,868,800	AA	AA	AA	AA	AA
RS1370543	85,870,310	BB	BB	BB	BB	BB
RS6757562	85,872,010	AA	AA	AA	AA	AA
RS13388951	85,872,080	AA	AB	BB	AA	AA
RS7609084	85,873,100	BB	AB	AA	BB	BB
RS4832201	85,874,180	AA	AB	BB	AA	AA
RS10180924	85,875,430	BB	AB	BB	AA	AA
RS1878901	85,876,910	AA	AB	AA	BB	BB
RS11684014	85,882,110	BB	BB	BB	BB	BB
RS6738956	85,883,550	AA	AA	BB	AA	AA
RS7576320	85,889,060	AA	BB	AA	BB	BB
RS13400884	85,893,330	BB	AA	AA	AA	AA
RS11679814	85,895,190	AA	AB	BB	BB	BB
RS10196236	85,914,900	BB	BB	BB	BB	BB
RS4832202	85,915,940	BB	AB	BB	AA	AA
RS13420465	85,916,550	AA	AB	BB	BB	BB
RS17026666	85,927,900	AA	AA	AA	AA	AA
RS10496320	85,931,990	AA	AA	AA	AA	AA
RS10496319	85,932,730	BB	BB	BB	BB	BB
RS1518990	85,932,850	AA	AA	AA	AA	AA
RS4553826	85,948,710	AA	AB	BB	AA	AA
RS4832015	85,950,320	AA	AB	BB	AA	AA
RS3810821	85,950,340	BB	BB	BB	BB	BB
RS6547638	85,954,520	AA	AB	BB	AA	AA
RS2030275	85,955,410	AA	AA	AA	AA	AA
RS17026728	85,955,530	AA	AA	AA	AA	AA

RS6721125	85,958,240	AA	AB	BB	AA	AA
RS12714154	85,971,470	AA	AB	AA	AB	AB
RS2438307	85,978,810	AA	AA	AA	AA	AA
RS2577777	85,987,830	BB	AB	AA	AB	AB
RS2679722	85,988,660	AA	AB	BB	AB	AB
RS2679723	85,989,120	BB	BB	BB	BB	BB
RS2679724	85,989,720	AA	AA	AA	AA	AA
RS2083170	85,992,620	AA	AB	AA	AB	AB
RS4292089	85,992,690	AA	AA	AA	AA	AA
RS2945825	85,993,310	AA	AB	BB	AB	AB
RS7593080	85,999,300	AA	AB	BB	AB	AB
RS2164878	86,007,370	BB	AB	BB	AB	AB
RS17026756	86,007,510	AA	AB	AA	AB	AB
RS6547647	86,008,780	AA	AB	AA	AB	AB
RS6710042	86,010,260	BB	BB	BB	BB	BB
RS4832226	86,010,490	BB	AB	BB	AB	AB
RS6547648	86,010,730	AA	AA	AA	AA	AA
RS11691934	86,018,950	AA	AB	BB	AB	AB
RS10167259	86,019,280	AA	AB	BB	AB	AB
RS2033770	86,021,340	BB	AB	AA	AB	AB
RS2366958	86,023,500	AA	BB	BB	BB	BB
RS1158729	86,026,150	BB	BB	BB	BB	BB
RS2366960	86,027,730	AA	AA	AA	AA	AA
RS6714363	86,032,480	BB	AB	BB	AB	AB
RS11891625	86,035,770	AA	AA	AA	AB	AB
RS10186005	86,036,940	BB	BB	BB	AB	AB
RS2099616	86,063,680	AA	AA	AA	AB	AB
RS4480982	86,068,570	AA	AA	AA	AB	AB
RS4346389	86,068,610	BB	BB	BB	AB	AB
RS7599498	86,079,890	AA	BB	AA	AB	AB
RS11127012	86,109,610	BB	AB	BB	AB	AB
RS7599656	86,119,140	AA	AA	AA	AA	AA
RS7572206	86,125,740	BB	BB	BB	BB	BB
RS1561328	86,126,270	AA	AB	AA	AA	AA
RS10184159	86,129,900	BB	AB	BB	BB	BB
RS311581	86,136,470	BB	AA	AA	AA	AA
RS311582	86,137,800	BB	AA	AA	AA	AA
RS311569	86,148,810	BB	AA	AA	AA	AA
RS311572	86,150,080	AA	BB	BB	BB	BB
RS2288117	86,158,630	BB	AA	AA	AA	AA
RS12328100	86,160,640	BB	AB	BB	BB	BB
RS17584578	86,161,140	BB	AB	BB	BB	BB
RS725046	86,181,700	AA	AB	BB	BB	BB
RS11127016	86,190,620	AA	AA	BB	BB	BB
RS1075622	86,191,440	BB	AB	AA	AA	AA
RS17026940	86,196,300	AA	AA	AA	AA	AA
RS17618119	86,196,820	BB	BB	AA	AB	AB
RS2303339	86,199,690	BB	AB	BB	BB	BB
RS1019592	86,208,220	AA	AB	BB	BB	BB
RS2241437	86,214,080	AA	AA	AA	AA	AA
RS2241436	86,214,320	AA	AB	BB	BB	BB

RS2241435	86,214,420	AA	AB	AA	AA	AA
RS10179466	86,228,580	AA	AB	AA	AA	AA
RS6547660	86,236,680	AA	AB	AA	AA	AA
RS7561589	86,238,740	BB	AB	BB	BB	BB
RS10191567	86,238,910	AA	BB	BB	BB	BB
RS3770066	86,245,860	AA	AB	AA	AA	AA
RS1424516	86,248,780	BB	AA	AA	AA	AA
RS7609008	86,253,390	BB	AB	BB	BB	BB
RS3821017	86,257,330	AA	BB	AA	AA	AA
RS17027011	86,260,060	AA	AB	AA	AA	AA
RS3770060	86,260,400	AA	AB	AA	AA	AA
RS965636	86,266,960	BB	BB	AA	AA	AA
RS10175330	86,267,620	AA	AB	AA	AA	AA
RS12996308	86,273,440	BB	AB	BB	BB	BB
RS11900161	86,280,740	AA	AB	BB	AA	AA
RS6547665	86,285,140	BB	AB	AA	BB	BB
RS3770059	86,289,580	AA	AB	AA	BB	BB
RS17438451	86,292,110	AA	AA	AA	BB	AB
RS17027032	86,292,580	AA	AA	AA	AA	AA
RS12329034	86,298,080	AA	AB	AA	AA	AA
RS7419344	86,304,660	AA	BB	BB	BB	BB
RS11127018	86,308,420	AA	AB	BB	BB	BB
RS13000103	86,309,000	BB	AB	AA	BB	BB
RS2042502	86,312,760	BB	BB	BB	AA	AA
RS4832258	86,318,030	AA	BB	AB	BB	BB
RS2278105	86,320,020	AA	AB	BB	AA	AA
RS13017143	86,320,060	BB	AB	AA	BB	BB
RS6714362	86,330,100	BB	AB	AA	BB	BB
RS1863053	86,330,980	BB	BB	BB	BB	BB
RS4832031	86,332,490	BB	BB	BB	AA	AA
RS4832261	86,338,010	BB	BB	BB	AA	AA
RS17510512	86,338,820	AA	AA	AA	AA	AA
RS17027110	86,341,620	AA	AA	AA	AA	AA
RS10204589	86,346,740	BB	AB	BB	AA	AA
RS12622200	86,352,100	BB	AB	AA	BB	BB
RS10520415	86,352,250	BB	AB	AA	BB	BB
RS11127024	86,358,960	AA	AA	AA	BB	BB
RS12617962	86,366,620	BB	BB	BB	BB	BB
RS17738096	86,367,410	AA	AB	BB	AA	AB
RS7587576	86,367,540	BB	BB	BB	BB	BB
RS13023154	86,370,500	BB	AB	AA	BB	BB
RS10188530	86,372,540	AA	AB	AA	AA	AA
RS1863065	86,376,950	AA	AB	BB	BB	BB
RS6760222	86,379,640	BB	BB	BB	BB	BB
RS13388621	86,386,470	BB	AB	BB	BB	BB
RS12478653	86,388,900	BB	AB	AA	BB	BB
RS6716825	86,391,580	BB	BB	BB	BB	BB
RS6704826	86,397,880	AA	AA	AA	AA	AA
RS11680025	86,398,850	AA	AA	AA	BB	BB
RS17027144	86,399,660	BB	BB	BB	BB	BB
RS7593666	86,404,350	AA	AA	AA	AA	AA

RS11675985	86,412,490	AA	AB	BB	BB	BB
RS1863052	86,412,870	AA	AB	BB	AA	AA
RS10210162	86,414,040	BB	AB	AA	AA	AA
RS6714317	86,416,010	BB	BB	BB	BB	BB
RS6718850	86,417,360	BB	AB	AA	AA	AA
RS12471787	86,420,210	BB	BB	AA	AA	AA
RS7589635	86,433,200	BB	BB	AA	AA	AA
RS7590671	86,437,140	AA	AA	AA	AA	AA
RS12714182	86,437,230	BB	BB	BB	BB	BB
RS13405197	86,437,760	BB	BB	AA	AA	AA
RS17027161	86,437,980	AA	AB	AA	AA	AA
RS4832278	86,442,400	BB	BB	BB	BB	BB
RS12151836	86,454,560	BB	BB	AA	AA	AA
RS7557127	86,463,210	BB	BB	BB	BB	BB
RS1033288	86,465,060	AA	AB	BB	BB	BB
RS6716701	86,465,650	AB	AB	AA	AA	AA
RS6547678	86,466,690	AA	AB	AA	AA	AA
RS6721311	86,466,980	AA	AB	AA	AA	AA
RS7609052	86,468,180	AA	AB	AA	AA	AA
RS13420491	86,468,600	BB	BB	AA	AA	AA
RS6547685	86,479,040	BB	BB	BB	BB	BB
RS1105862	86,480,480	BB	BB	BB	BB	BB
RS4832282	86,481,170	BB	BB	AA	AA	AA
RS12617075	86,483,700	BB	BB	AA	AA	AA
RS1113169	86,489,020	AA	AB	AA	AA	AA
RS4832285	86,498,180	BB	BB	BB	BB	BB
RS17027257	86,507,210	BB	AB	BB	BB	BB
RS10865482	86,508,400	BB	BB	AA	AA	AA
RS11127037	86,510,410	BB	AB	BB	BB	BB
RS2030259	86,537,150	AA	AA	AA	AA	AA
RS10496321	86,537,220	AA	AB	AA	AA	AA
RS11127042	86,551,710	AA	AA	AA	AA	AA
RS17027280	86,586,800	AA	AA	BB	BB	BB
RS4832294	86,589,870	BB	BB	BB	BB	BB
RS11127043	86,591,830	BB	BB	AA	AA	AA
RS17027287	86,609,600	AA	AA	AA	AA	AA
RS1036940	86,609,720	AA	AA	AA	AA	AA
RS12987172	86,643,100	AA	AA	BB	BB	BB
RS12328637	86,677,700	AA	AA	BB	BB	BB
RS10520428	86,693,040	BB	AB	BB	BB	BB
RS308907	86,695,290	AA	AA	AA	AA	AA
RS10451632	86,697,430	AA	AA	AA	AA	AA
RS308901	86,700,000	AA	AA	AA	AA	AA
RS308910	86,709,370	AA	AA	BB	BB	BB
RS308911	86,709,490	AA	AA	AA	AA	AA
RS2044234	86,711,490	AB	BB	BB	BB	BB
RS308916	86,713,420	AA	AA	BB	AB	AB
RS1370452	86,726,100	AA	AB	AA	AA	AA
RS7420475	86,726,180	AA	AA	AA	AA	AA
RS4402760	86,726,340	BB	BB	BB	BB	BB
RS6547701	86,727,890	AA	AB	AA	AA	AA

RS9808243	86,729,060	AA	AB	AA	AA	AA
RS12613610	86,751,010	BB	AB	BB	BB	BB
RS10201581	86,778,720	AA	AA	AA	AA	AA
RS2612952	86,790,940	AA	AA	AA	AA	AA
RS13406312	86,823,920	AA	AA	AA	AA	AA
RS4832311	86,832,410	AA	AB	BB	BB	BB
RS1878109	86,836,380	AA	AA	BB	BB	BB
RS1049457	86,856,540	BB	BB	AA	AA	AA
RS938487	86,863,000	AA	AB	AA	AA	AA
RS1515950	86,874,330	AA	AA	AA	AA	AA
RS7577266	86,874,960	BB	BB	BB	BB	BB
RS13023213	86,875,460	AA	AA	AA	AA	AA
RS1005834	86,877,270	AA	AB	BB	AB	AB
RS12477946	86,897,300	AA	AA	AA	AA	AA
RS13001400	86,902,220	BB	BB	BB	BB	BB
RS4488661	86,906,660	AA	AA	AA	AA	AA
RS6749703	86,908,760	BB	BB	BB	BB	BB
RS4240205	86,910,320	AA	AB	BB	AB	AB
RS10189173	86,924,860	AA	AA	AA	AA	AA
RS6724300	86,979,760	BB	BB	BB	AB	AB
RS10203656	87,132,390	BB	AB	AA	BB	BB
RS2612908	87,270,590	AA	AB	AA	AA	AA
RS1518713	87,373,860	AA	AB	BB	AA	AA
RS4971998	87,539,110	AA	BB	BB	AB	AB
RS6547739	87,732,730	AA	AB	AA	AB	AB
RS1710447	87,741,700	AA	AA	BB	AA	AA
RS1441649	88,076,340	AA	AA	AB	AB	AB
RS10197574	88,077,080	BB	BB	AA	AA	AA
RS12714205	88,077,460	BB	BB	AA	AA	AA
RS6735537	88,092,100	AA	AA	AA	AA	AA
RS11684204	88,118,530	AA	AA	BB	BB	BB
RS6547747	88,121,120	AA	AA	BB	BB	BB
RS4972168	88,132,000	BB	BB	AA	AA	AA
RS11127085	88,143,860	AA	AA	AA	AA	AA
RS2919874	88,150,910	AA	AA	BB	BB	BB
RS11682584	88,152,410	AA	AA	AA	AA	AA
RS2919878	88,152,560	BB	AB	AA	AA	AA
RS13387249	88,154,900	AA	AA	AA	AA	AA
RS2099825	88,159,880	BB	AB	AA	BB	BB
RS9309643	88,160,720	BB	BB	BB	BB	BB
RS2970896	88,164,250	AB	AB	AA	AA	AA
RS2919857	88,169,050	BB	BB	AA	BB	BB
RS2919854	88,170,880	BB	AA	AA	AA	AA
RS2970909	88,176,220	AA	BB	BB	AA	AA
RS2970911	88,176,940	AA	BB	BB	AA	AA
RS10172567	88,178,700	AA	AA	AA	AA	AA
RS2919880	88,179,030	BB	AA	AA	AA	AA
RS2970923	88,181,120	BB	AB	AA	AA	AA
RS2970920	88,187,380	BB	BB	AA	BB	BB
RS10192400	88,190,300	AA	AB	BB	BB	BB
RS4972176	88,190,480	AA	AB	AA	BB	BB

RS2919863	88,190,600	AA	AB	AA	BB	BB
RS13386552	88,197,550	BB	BB	BB	BB	BB
RS894194	88,199,660	AA	AB	AA	AA	AA
RS1545224	88,204,930	BB	BB	AA	BB	BB
RS2241883	88,205,180	AA	AB	AA	AA	AA
RS13400963	88,208,060	BB	BB	BB	BB	BB
RS2970924	88,214,420	BB	AB	BB	AA	AA
RS7581571	88,216,350	AA	AA	AA	AA	AB
RS4386315	88,216,940	AA	AB	AA	BB	AB
RS7420937	88,218,280	AA	AA	AA	AA	AA
RS4325755	88,225,590	BB	BB	AA	BB	BB
RS10168005	88,226,930	AA	AB	AA	BB	BB
RS12463835	88,227,730	AA	AB	AA	BB	BB
RS10173112	88,244,270	AA	AB	AA	BB	BB
RS10185660	88,244,350	BB	BB	BB	BB	BB
RS9636487	88,246,080	BB	BB	BB	BB	BB
RS11683461	88,252,910	AA	AA	AA	AA	AA
RS2139100	88,255,490	BB	AB	BB	AA	AA
RS2292868	88,256,160	BB	BB	BB	BB	BB
RS12621922	88,276,110	AA	AA	AA	AA	AA
RS1878813	88,279,390	BB	BB	BB	AA	AB
RS17435433	88,290,730	BB	AB	BB	AA	AA
RS6547762	88,296,880	AA	AB	AA	BB	BB
RS10175119	88,300,920	BB	BB	BB	BB	BB
RS2363731	88,302,910	AA	AB	AA	AA	AA
RS7425670	88,303,140	AA	AB	AA	AA	AA
RS6756194	88,303,240	AA	AB	AA	BB	BB
RS4487093	88,304,020	AA	AB	AA	BB	BB
RS10173160	88,304,290	AB	AB	AA	BB	BB
RS17037827	88,306,050	BB	BB	BB	BB	BB
RS4260241	88,307,080	AA	AA	AA	AA	AA
RS4972193	88,307,680	AA	AB	AA	AA	AA
RS6755212	88,308,260	BB	BB	BB	BB	BB
RS12995491	88,308,530	AA	BB	AA	BB	BB
RS11694318	88,316,440	AA	AB	AA	AA	AA
RS1914732	88,322,430	AA	BB	BB	AA	AA
RS2090562	88,330,060	BB	AB	BB	BB	BB
RS10178983	88,346,360	AA	AA	AA	AA	AA
RS6729287	88,347,250	BB	AB	BB	BB	BB
RS6547770	88,349,990	AA	AB	AA	AA	AA
RS6728787	88,355,940	BB	AB	BB	BB	BB
RS7592389	88,364,700	AA	AB	AA	AA	AA
RS7595764	88,371,250	AA	AA	AA	AA	AA
RS6750741	88,372,120	AA	AA	AA	AA	AA
RS1258391	88,396,340	BB	BB	BB	BB	BB
RS17838451	88,398,290	AA	AB	AA	AA	AA
RS1258411	88,399,210	BB	AA	BB	BB	BB
RS1258408	88,400,610	BB	BB	BB	BB	BB
RS17037727	88,410,260	AA	AA	AA	AA	AA
RS1713941	88,410,470	AA	AA	AA	AA	AA
RS1258419	88,412,290	AA	AA	BB	BB	BB

RS1258418	88,412,380	BB	BB	AA	AA	AA
RS1713932	88,415,280	AA	AA	BB	BB	BB
RS1659248	88,416,090	AA	AB	BB	BB	BB
RS1258425	88,432,340	BB	BB	AA	AA	AA
RS1659258	88,440,700	BB	AB	AA	AA	AA
RS7559276	88,451,270	AA	AB	AA	AA	AA
RS6726237	88,460,930	AA	BB	AA	AA	AA
RS10221815	88,479,080	BB	AA	BB	BB	BB
RS10221774	88,480,790	BB	BB	BB	BB	BB
RS10221875	88,481,280	AA	AA	AA	AA	AA
RS13425454	88,491,080	BB	BB	BB	BB	BB
RS6704818	88,498,560	AA	AA	AA	AA	AA
RS6749685	88,502,260	BB	BB	BB	BB	BB
RS6721576	88,519,620	AA	AA	BB	AA	AA
RS6722564	88,520,410	AA	AB	AA	BB	BB
RS6737701	88,520,450	BB	BB	AA	BB	BB
RS6725764	88,520,760	AA	AB	BB	AA	AA
RS10203213	88,539,710	AA	AB	BB	AA	AA
RS6739967	88,539,890	AA	AA	AA	AA	AA
RS11690974	88,563,910	AA	BB	AA	AA	AA
RS11677224	88,570,930	AA	BB	AA	AA	AA
RS12621362	88,585,000	BB	BB	BB	BB	BB
RS11683161	88,589,120	AA	BB	AA	AA	AA
RS11127100	88,612,080	BB	AA	BB	BB	BB
RS6738571	88,612,220	AA	BB	AA	AA	AA
RS11900268	88,624,460	BB	BB	BB	BB	BB
RS12623311	88,626,260	BB	AA	BB	BB	BB
RS4444527	88,627,740	BB	BB	BB	BB	BB
RS4449134	88,628,080	BB	BB	BB	BB	BB
RS6739095	88,661,180	AA	AB	BB	AA	AA
RS1913671	88,681,000	BB	AB	AA	BB	BB
RS10187483	88,684,880	AA	AA	AA	AA	AA
RS867014	88,700,820	AA	AA	AA	AA	AA
RS11684404	88,705,740	AA	AB	BB	AA	AA
RS2364564	88,712,580	AA	AB	BB	AA	AA
RS2365106	88,729,220	BB	AB	AA	BB	BB
RS13426600	88,731,040	BB	BB	BB	BB	BB
RS17037607	88,737,230	BB	BB	BB	BB	BB
RS2365107	88,754,620	AA	AA	AA	AA	AA
RS13405965	88,754,870	AA	AA	AA	AA	AA
RS7421753	88,757,640	AA	AA	AA	AA	AA
RS2176053	88,758,110	BB	BB	BB	BB	BB
RS13411467	88,758,220	BB	BB	BB	BB	BB
RS6747173	88,767,360	AA	AA	AA	AA	AA
RS6718735	88,767,490	AA	AA	AA	AA	AA
RS17037578	88,771,380	BB	BB	BB	BB	BB
RS17838439	88,788,400	AA	AA	AA	AA	AA
RS6742845	88,796,280	BB	BB	BB	BB	BB
RS7596685	88,807,890	AB	AA	AA	AA	AA
RS17037560	88,817,800	AA	AA	AA	AA	AA
RS17037556	88,820,910	AA	AA	AA	AA	AA

RS4972012	88,826,700	BB	BB	BB	BB	BB
RS7572313	88,861,510	BB	BB	BB	BB	BB
RS2848122	88,888,300	BB	AB	BB	BB	BB
RS2628474	88,890,860	BB	AB	BB	BB	BB
RS2628473	88,890,890	AA	AB	AA	AA	AA
RS6547793	88,897,510	AA	AA	AA	AA	AA
RS1809526	88,906,600	BB	BB	BB	BB	BB
RS17838437	88,911,120	AA	AA	AA	AA	AA
RS4972036	89,395,620	AA	AA	AA	AA	AA
RS4972121	89,400,110	AA	BB	AA	AA	AA
RS11127124	89,401,950	BB	AA	BB	BB	BB
RS4101813	89,402,220	BB	BB	BB	BB	BB
RS2195078	89,682,170	BB	BB	BB	AA	AA
RS17704088	89,682,550	BB	BB	BB	BB	BB
RS2847840	89,714,800	AA	BB	BB	BB	BB
RS859750	89,753,410	AA	BB	BB	BB	BB
RS842160	89,879,380	AA	AA	AA	AA	AA
RS4005764	90,982,620	AA	AA	AA	AA	AA
RS1901291	91,091,700	BB	BB	BB	BB	BB
RS9753235	91,159,270	AA	AA	AA	AA	AA
RS232162	91,177,840	AA	AA	AA	AA	AA
RS10204030	94,735,980	BB	AA	AA	BB	BB
RS12717791	94,784,020	AA	BB	BB	AA	AA
RS1809636	94,785,420	BB	BB	BB	AA	AA
RS13424824	94,917,420	BB	BB	BB	BB	BB
RS7587312	94,938,730	BB	BB	BB	AA	AA
RS10210891	94,944,590	BB	BB	BB	AA	AA
RS6710440	94,956,590	BB	BB	BB	BB	BB
RS7604922	95,039,200	BB	BB	BB	BB	BB
RS4241315	95,050,830	AA	AA	AA	AA	AA
RS2320619	95,098,350	AA	AA	AA	AA	AA
RS4854244	95,101,410	AA	AA	AA	AA	AA
RS3112996	95,176,100	AA	AA	AA	AA	AA
RS2320625	95,226,460	BB	BB	BB	BB	BB
RS13385838	95,231,980	BB	BB	BB	BB	BB
RS7589426	95,266,900	AA	AA	AA	AA	AA
RS7563350	95,294,940	BB	BB	BB	BB	BB
RS1808455	95,329,020	BB	BB	BB	BB	BB
RS6715813	95,334,100	AA	AA	AA	AA	AA
RS3755523	95,338,540	AA	AA	AA	AA	AA
RS889851	95,344,060	BB	BB	BB	BB	BB
RS2113417	95,363,580	BB	BB	BB	BB	BB
RS9678800	95,373,050	BB	BB	BB	BB	BB
RS3772031	95,378,620	BB	BB	BB	BB	BB
RS950320	95,392,890	BB	BB	BB	BB	BB
RS12996789	95,424,970	AA	AA	AA	AA	AA
RS2320170	95,603,500	BB	BB	BB	BB	BB
RS10176771	95,697,520	BB	BB	BB	BB	BB
RS11676901	95,734,300	AA	AA	AA	AA	AA
RS17119922	95,743,980	AA	AA	AA	AA	AA
RS1989620	95,749,460	AA	AA	AA	AA	AA

RS4907276	95,754,830	BB	BB	BB	BB	BB
RS4907280	95,765,860	AA	AA	AA	AA	AA
RS1808357	95,785,150	BB	AA	BB	AA	AA
RS1813381	95,786,660	BB	BB	BB	BB	BB
RS1917890	96,035,730	AA	AA	AA	AA	AA
RS2579516	96,048,600	AA	BB	BB	BB	BB
RS2579551	96,102,310	BB	AA	BB	AA	AA
RS1168965	96,151,620	BB	BB	BB	BB	BB
RS1168968	96,189,090	AA	AA	AA	AA	AA
RS4907301	96,200,240	AA	AA	AA	BB	BB
RS3770239	96,281,970	AA	AA	AA	AA	AA
RS1044594	96,301,020	BB	BB	AA	BB	BB
RS17479466	96,326,580	AA	AA	BB	AA	AA
RS772178	96,327,410	BB	BB	BB	BB	BB
RS17119410	96,328,220	AA	AA	AA	AA	AA
RS772173	96,364,120	BB	AA	AA	BB	BB
RS3731935	96,383,780	BB	BB	AA	BB	BB
RS687950	96,433,340	BB	AA	AA	BB	BB
RS584811	96,433,610	BB	AA	BB	BB	BB
RS12614696	96,482,690	AA	BB	AA	AA	AA
RS6576972	96,516,180	AA	BB	BB	BB	BB
RS2118836	96,526,700	AA	AA	BB	BB	BB
RS2279049	96,526,900	AA	AA	AA	AA	AA
RS1866444	96,535,860	BB	BB	BB	BB	BB
RS2579503	96,540,360	BB	BB	BB	BB	BB
RS4907241	96,605,660	BB	BB	AA	AA	AA
RS12469420	96,607,030	BB	BB	AA	AA	AA
RS2314109	96,607,260	AA	BB	BB	BB	BB
RS7604736	96,607,890	AA	BB	AA	AA	AA
RS17119533	96,645,140	AA	AA	AA	AA	AA
RS2280355	96,730,030	AA	AA	AA	AA	AA
RS2280356	96,730,050	BB	BB	AA	BB	BB
RS6746896	96,774,670	BB	BB	BB	AA	AA
RS2678381	96,804,990	AA	BB	BB	BB	BB
RS7608661	96,823,650	AA	AA	BB	BB	BB
RS7580942	96,832,000	AA	AA	BB	AA	AA
RS1878656	96,842,070	AA	AA	AA	AA	AA
RS17119598	96,850,040	BB	BB	AA	BB	BB
RS7594727	96,853,600	BB	BB	AA	BB	BB
RS9948	96,864,530	BB	BB	AA	BB	BB
RS7587149	96,866,530	BB	BB	BB	BB	BB
RS7566053	96,893,120	AA	AA	AA	AA	AA
RS17119643	96,917,020	BB	BB	BB	BB	BB
RS12613171	96,917,470	AA	BB	AA	AA	AA
RS4907206	96,924,640	BB	AA	BB	BB	BB
RS10182965	96,926,280	AA	BB	AA	AA	AA
RS11676858	96,961,300	AA	AA	AA	BB	BB
RS13403225	96,964,550	BB	BB	BB	AA	AA
RS7593754	96,971,270	AA	AA	BB	AA	AA
RS6707255	96,975,940	BB	BB	BB	BB	BB
RS1623881	96,977,470	BB	AA	BB	BB	BB

RS1257022	96,978,660	BB	BB	BB	AA	AA
RS1730127	96,986,140	BB	BB	AA	AA	AA
RS4586672	96,991,960	BB	AA	BB	BB	BB
RS1796032	96,992,260	BB	AA	BB	BB	BB
RS12612567	96,992,390	AA	AA	AA	AA	AA
RS1257007	96,999,040	BB	BB	BB	AA	AA
RS1257027	97,019,610	AA	BB	AA	AA	AA
RS1257026	97,019,690	AA	AA	AA	BB	BB
RS17636359	97,024,340	BB	BB	BB	BB	BB
RS1256999	97,043,660	BB	BB	BB	AA	AA
RS1256995	97,047,170	BB	BB	BB	BB	BB
RS1256987	97,058,280	BB	BB	BB	AA	AA
RS1148600	97,067,010	BB	BB	BB	AA	AA
RS1257006	97,072,340	BB	BB	BB	AA	AA
RS1257089	97,117,310	BB	BB	BB	AA	AA
RS1257084	97,121,230	BB	BB	BB	AA	AA
RS1257101	97,122,680	BB	BB	BB	AA	AA
RS1257097	97,128,180	AA	AA	AA	BB	BB
RS11895531	97,129,290	AA	AA	AA	AA	AA
RS7582819	97,237,050	AA	AA	AA	AA	AA
RS4264593	97,379,950	BB	AA	BB	AA	AA
RS10199493	97,385,000	AA	AA	AA	AA	AA
RS6707273	97,386,660	AA	AA	AA	AA	AA
RS3906948	97,591,870	BB	AA	AA	BB	BB
RS1375436	97,637,310	BB	BB	AA	AA	AA
RS1042705	97,640,960	BB	BB	AA	AA	AA
RS2053824	97,648,060	BB	AA	AA	AA	AA
RS11123849	97,652,660	AA	BB	BB	BB	BB
RS10496323	97,656,270	BB	BB	BB	AA	AA
RS17028568	97,672,230	BB	BB	BB	BB	BB
RS11893601	97,681,340	BB	AA	AA	AA	AA
RS11680855	97,684,120	BB	BB	BB	AA	AA
RS11894651	97,684,200	AA	BB	BB	BB	BB
RS2290127	97,685,460	AA	AA	AA	AA	AA
RS7425883	97,697,950	BB	BB	BB	AA	AA
RS6711959	97,705,660	AA	AA	BB	AA	AA
RS13033383	97,705,940	AA	AA	AA	AA	AA
RS895437	97,716,780	BB	BB	BB	BB	BB
RS1466020	97,731,680	BB	BB	BB	BB	BB
RS17021963	97,734,100	BB	BB	BB	BB	BB
RS930607	97,737,890	BB	BB	BB	BB	BB
RS6543088	97,738,710	BB	AA	AA	BB	BB
RS6875	97,739,410	BB	AA	AA	BB	BB
RS5865	97,739,440	AA	BB	BB	AA	AA
RS2305142	97,742,150	BB	AA	BB	BB	BB
RS6760008	97,744,100	AA	BB	BB	AA	AA
RS3749081	97,749,420	AA	AA	AA	AA	AA
RS6738389	97,753,160	AA	AA	AA	AA	AA
RS6721323	97,753,610	AA	BB	AA	AA	AA
RS3821053	97,758,320	BB	AA	BB	BB	BB
RS17022289	97,769,940	AA	AA	AA	AA	AA

RS17022373	97,773,600	BB	BB	BB	BB	BB
RS17489454	97,773,780	AA	AA	AA	AA	AA
RS2293679	97,779,500	AA	BB	AA	AA	AA
RS10188713	97,782,560	AA	BB	AA	AA	AA
RS7558931	97,805,260	BB	BB	BB	BB	BB
RS7576509	97,815,170	BB	BB	BB	BB	BB
RS17424787	97,815,620	AA	AA	AA	AA	AA
RS11690687	97,828,260	AA	BB	AA	AA	AA
RS10520549	97,866,240	AA	AA	AA	AA	AA
RS13008503	97,869,650	BB	AA	BB	BB	BB
RS10191101	97,900,500	BB	AA	AA	BB	BB
RS1922280	97,904,540	AA	AA	AA	AA	AA
RS1503202	97,915,290	AA	BB	AA	AA	AA
RS11886417	97,941,960	AA	BB	BB	AA	AA
RS7598116	97,942,320	BB	BB	BB	BB	BB
RS9308873	97,994,020	BB	BB	BB	BB	BB
RS17025744	98,002,560	AA	BB	AA	AA	AA
RS17025778	98,003,940	AA	BB	AA	AA	AA
RS10460482	98,010,610	AA	BB	BB	BB	BB
RS7574926	98,022,420	BB	AA	BB	BB	BB
RS4499469	98,062,380	BB	BB	BB	BB	BB
RS2034547	98,068,460	BB	BB	BB	BB	BB
RS2122753	98,083,530	BB	BB	BB	BB	BB
RS6725198	98,085,500	BB	BB	BB	BB	BB
RS10204649	98,098,370	AA	AA	AA	BB	BB
RS6733649	98,123,340	BB	BB	BB	AA	AA
RS13033420	98,124,750	AA	BB	AA	BB	BB
RS7598069	98,127,820	BB	AA	BB	BB	BB
RS17426403	98,129,590	BB	BB	BB	BB	BB
RS13424156	98,129,720	BB	BB	BB	BB	BB
RS6744080	98,131,070	AA	BB	AA	AA	AA
RS6543330	98,139,940	BB	BB	BB	BB	BB
RS17492354	98,143,290	AA	BB	BB	AA	AA
RS13399675	98,155,780	AA	AA	AA	AA	AA
RS4851884	98,162,220	AA	BB	BB	BB	BB
RS17028309	98,166,320	AA	AA	AA	AA	AA
RS1466156	98,170,740	AA	AA	AA	AA	AA
RS2122755	98,174,180	BB	BB	BB	BB	BB
RS4851915	98,175,060	BB	BB	BB	BB	BB
RS7579916	98,177,500	BB	BB	BB	BB	BB
RS10187244	98,182,440	BB	AA	AA	AA	AA
RS17028800	98,190,610	BB	BB	BB	BB	BB
RS4132466	98,202,390	AA	AA	AA	AA	AA
RS4632400	98,205,640	BB	AA	AA	AA	AA
RS13430738	98,205,800	AA	AA	AA	AA	AA
RS12712252	98,212,840	AA	AA	AA	AA	AA
RS11900430	98,213,390	AA	AA	AA	AA	AA
RS6543419	98,220,820	AA	AA	AA	AA	AA
RS12470615	98,224,740	BB	AA	AA	AA	AA
RS10176437	98,254,820	BB	BB	BB	BB	BB
RS4851957	98,256,390	BB	BB	BB	BB	BB

RS17493153	98,257,530	AA	AA	AA	AA	AA
RS7606070	98,264,470	AA	BB	BB	BB	BB
RS17493655	98,264,660	AA	BB	BB	BB	BB
RS6731704	98,283,000	AA	AA	AA	AA	AA
RS10176056	98,284,580	BB	BB	BB	BB	BB
RS3731659	98,288,260	AA	AA	AA	AA	AA
RS11124167	98,298,160	AA	AA	AA	AA	AA
RS10198592	98,302,410	BB	BB	BB	BB	BB
RS7571110	98,315,910	AA	BB	BB	AA	AA
RS13394706	98,319,830	AA	AA	AA	AA	AA
RS11123677	98,355,500	AA	BB	AA	BB	BB
RS10865015	98,355,640	AA	AA	BB	AA	AA
RS3769755	98,355,850	BB	BB	BB	BB	BB
RS3769754	98,356,130	AA	BB	AA	BB	BB
RS937725	98,360,850	BB	BB	BB	BB	BB
RS4146045	98,361,070	AA	AA	AA	AA	AA
RS3769745	98,367,830	AA	AA	AA	AA	AA
RS6743554	98,372,030	BB	AA	BB	BB	BB
RS2271041	98,374,780	BB	BB	BB	BB	BB
RS17030716	98,375,610	AA	AA	AA	AA	AA
RS10184555	98,378,690	AA	AA	AA	AA	AA
RS17031139	98,402,960	BB	BB	BB	BB	BB
RS11884501	98,430,180	BB	BB	BB	BB	BB
RS3769740	98,435,640	BB	BB	BB	BB	BB
RS17031905	98,437,340	AA	AA	AA	AA	AA
RS17444095	98,442,250	AA	AA	AA	AA	AA
RS3769739	98,443,460	BB	BB	BB	BB	BB
RS17032120	98,449,370	BB	BB	BB	BB	BB
RS12617721	98,450,320	BB	BB	BB	BB	BB
RS11901568	98,495,060	BB	BB	BB	BB	BB
RS6753380	98,495,470	AA	AA	AA	AA	AA
RS17504774	98,502,060	BB	BB	BB	BB	BB
RS2278214	98,502,170	BB	BB	BB	BB	BB
RS17504837	98,502,560	BB	BB	BB	BB	BB
RS17033136	98,515,840	BB	BB	BB	BB	BB
RS2278210	98,516,630	AA	AA	AA	AA	AA
RS3769714	98,517,230	BB	BB	BB	AA	AA
RS7559530	98,541,340	AA	AA	AA	AA	AA
RS10201079	98,559,080	AA	AA	AA	BB	BB
RS17033813	98,562,910	BB	BB	BB	BB	BB
RS3754875	98,569,180	AA	AA	AA	AA	AA
RS6736755	98,570,860	BB	BB	BB	BB	BB
RS6542833	98,574,640	BB	BB	BB	AA	AA
RS4851145	98,589,340	BB	BB	BB	BB	BB
RS3754872	98,590,360	AA	AA	AA	AA	AA
RS12618769	98,606,360	BB	BB	BB	BB	BB
RS17513621	98,612,990	AA	AA	AA	AA	AA
RS13021375	98,620,460	AA	AA	AA	AA	AA
RS17021689	98,622,340	BB	BB	BB	BB	BB
RS3754870	98,638,490	AA	AA	AA	AA	AA
RS17448420	98,664,310	AA	AA	AA	AA	AA

RS2309434	98,672,940	AA	AA	AA	AA	AA
RS882164	98,684,120	AA	AA	AA	AA	AA
RS13425789	98,688,160	AA	AA	AA	AA	AA
RS11123747	98,695,190	AA	BB	BB	BB	BB
RS4851150	98,705,550	BB	AA	AA	AA	AA
RS10496330	98,706,560	BB	AA	BB	BB	BB
RS17021908	98,728,620	AA	AA	AA	AA	AA
RS6706340	98,736,280	AA	AA	BB	BB	BB
RS6756691	98,742,230	AA	AA	AA	AA	AA
RS7573844	98,750,460	BB	AA	AA	AA	AA
RS925886	98,751,460	AA	AA	AA	AA	AA
RS35979410	98,770,900	BB	BB	BB	BB	BB
RS1540785	98,798,240	BB	BB	BB	BB	BB
RS2203432	98,801,190	BB	BB	AA	AA	AA
RS6733135	98,810,770	AA	BB	BB	BB	BB
RS12622681	98,828,540	BB	BB	AA	BB	BB
RS11900176	98,829,970	BB	AA	BB	BB	BB
RS981602	98,830,700	BB	BB	AA	AA	AA
RS6542839	98,830,960	AA	AA	BB	BB	BB
RS12616866	98,831,100	AA	AA	AA	AA	AA
RS6757098	98,837,830	BB	BB	AA	AA	AA
RS4851165	98,851,860	AA	AA	AA	AA	AA
RS868921	98,858,200	AA	AA	AA	AA	AA
RS4851168	98,863,190	BB	BB	BB	BB	BB
RS17022036	98,863,360	BB	BB	BB	BB	BB
RS17022040	98,866,900	BB	BB	BB	BB	BB
RS17022042	98,867,140	BB	BB	BB	BB	BB
RS12614153	98,868,920	AA	AA	AA	AA	AA
RS4851170	98,870,750	AA	AA	BB	BB	BB
RS17022046	98,875,950	BB	BB	BB	BB	BB
RS925887	98,881,100	AA	AA	BB	BB	BB
RS6731293	98,889,820	AA	AA	AA	AA	AA
RS17022066	98,897,360	AA	AA	AA	AA	AA
RS6542843	98,900,160	BB	BB	AA	AA	AA
RS6724477	98,903,500	AA	AA	BB	BB	BB
RS6732579	98,926,670	BB	AA	AA	AA	AA
RS7579984	98,929,730	BB	BB	BB	BB	BB
RS1453564	98,930,860	BB	BB	AA	AA	AA
RS2309519	98,936,820	BB	BB	BB	BB	BB
RS2309520	98,936,910	AA	AA	BB	BB	BB
RS6542847	98,939,900	BB	BB	AA	BB	BB
RS988825	98,942,140	BB	BB	BB	BB	BB
RS6740054	98,943,060	BB	BB	AA	AA	AA
RS12612688	98,979,520	BB	BB	AA	AA	AA
RS4850895	99,010,900	AA	AA	AA	AA	AA
RS12620997	99,063,240	AA	AA	AA	AA	AA
RS1563107	99,067,710	AA	AA	BB	BB	BB
RS12613624	99,099,180	BB	BB	AA	AA	AA
RS1133977	99,108,420	BB	BB	AA	AA	AA
RS11695379	99,108,590	AA	AA	BB	BB	BB
RS13428547	99,110,690	BB	BB	BB	BB	BB

RS4851187	99,112,290	BB	BB	AA	AA	AA
RS3791211	99,145,170	AA	AA	AA	AA	AA
RS2309581	99,150,720	BB	BB	AA	AA	AA
RS2309582	99,150,740	BB	BB	BB	BB	BB
RS11689265	99,165,700	AA	AA	BB	BB	BB
RS17022330	99,169,100	AA	AA	AA	AA	AA
RS10865030	99,171,070	AA	BB	BB	BB	BB
RS7586635	99,194,170	BB	AA	AA	AA	AA
RS7603462	99,214,320	AA	BB	BB	BB	BB
RS7579999	99,224,100	AA	BB	BB	BB	BB
RS995617	99,224,340	BB	BB	BB	BB	BB
RS4851196	99,224,990	BB	AA	BB	BB	BB
RS17022433	99,240,090	AA	BB	AA	BB	BB
RS4850899	99,243,900	AA	AA	BB	AA	AA
RS1922625	99,280,430	BB	AA	AA	AA	AA
RS4851201	99,296,250	BB	AA	AA	AA	AA
RS12623620	99,342,300	BB	BB	BB	BB	BB
RS2309617	99,342,720	BB	AA	BB	AA	AA
RS3087388	99,385,990	AA	AA	AA	AA	AA
RS13402784	99,398,880	BB	AA	AA	AA	AA
RS7602535	99,406,100	AA	AA	AA	AA	AA
RS9941566	99,406,330	BB	BB	BB	BB	BB
RS3792151	99,408,080	BB	AA	AA	AA	AA
RS28382892	99,419,730	BB	BB	BB	BB	BB
RS10186223	99,424,860	BB	AA	AA	AA	AA
RS3792138	99,427,430	BB	BB	BB	BB	BB
RS7594838	99,433,980	BB	BB	AA	BB	BB
RS7567674	99,434,010	AA	AA	BB	AA	AA
RS7585019	99,443,760	BB	AA	BB	AA	AA
RS6542882	99,464,050	AA	AA	BB	AA	AA
RS6714244	99,471,740	BB	BB	AA	BB	BB
RS6715321	99,475,430	BB	AA	BB	AA	AA
RS11887574	99,475,680	AA	AA	BB	AA	AA
RS13384883	99,489,020	BB	BB	AA	BB	BB
RS1901284	99,512,470	BB	BB	AA	AB	AB
RS17765112	99,515,770	AA	AA	BB	AB	AB
RS4850905	99,518,800	AA	AA	AA	AB	AB
RS3792119	99,538,510	AA	AA	AA	AA	AA
RS11890008	99,540,660	AA	AA	AA	AA	AA
RS17022781	99,540,970	AA	AA	AA	AA	AA
RS10179214	99,541,150	BB	BB	BB	BB	BB
RS9808393	99,564,070	BB	BB	BB	BB	BB
RS12998444	99,570,480	AA	AA	AA	AB	AB
RS12712049	99,575,260	AA	AA	BB	BB	BB
RS10210038	99,588,510	AA	AA	AA	AA	AA
RS11123789	99,590,130	BB	AA	AA	AA	AA
RS6718629	99,605,180	AA	AA	AA	AA	AA
RS1568785	99,612,580	BB	AA	BB	AB	AB
RS2177541	99,613,360	AA	BB	AA	AA	AA
RS3811556	99,627,290	BB	BB	BB	AB	AB
RS17022829	99,627,880	BB	BB	AB	AB	AB

RS17022834	99,629,230	AA	AA	AA	AB	AB
RS6719933	99,630,410	AB	AB	AB	AB	AB
RS6745568	99,630,580	BB	AA	BB	BB	BB
RS17022852	99,635,740	BB	BB	AA	AB	AB
RS12712054	99,637,130	BB	AA	BB	BB	BB
RS6728983	99,640,330	BB	AA	AA	AB	AB
RS6749801	99,640,480	AA	AA	AA	AA	AA
RS2030693	99,654,380	AA	AB	AA	AA	AA
RS17022868	99,657,710	BB	BB	BB	BB	BB
RS6542886	99,657,850	AA	AA	BB	BB	BB
RS7560182	99,659,540	AA	AA	BB	BB	BB
RS7561009	99,660,260	AA	AA	AA	AA	AA
RS10189976	99,662,070	AA	AA	BB	BB	BB
RS17022882	99,667,390	AA	AA	AA	AB	AB
RS6728654	99,670,150	AA	AA	AA	AA	AA
RS6730914	99,675,610	BB	BB	BB	AB	AB
RS17022916	99,679,390	BB	BB	BB	BB	BB
RS12712058	99,691,200	BB	BB	AA	AB	AB
RS17022970	99,705,380	AA	AA	AA	AA	AA
RS10496339	99,713,300	BB	BB	BB	BB	BB
RS1404177	99,725,360	BB	BB	AA	BB	BB
RS2091150	99,741,710	BB	BB	BB	BB	BB
RS6721652	99,743,750	AA	AA	AA	AA	AA
RS6542892	99,749,390	BB	BB	BB	BB	BB
RS17023084	99,750,190	AB	AB	BB	AB	AB
RS4851231	99,752,100	BB	BB	AA	BB	BB
RS17023090	99,755,220	AA	AA	AA	AA	AA
RS6755935	99,762,970	AA	AA	BB	AA	AA
RS1366761	99,767,180	AA	AA	AA	AA	AB
RS6719359	99,770,660	BB	BB	BB	BB	BB
RS7604438	99,772,410	AA	AA	AA	AA	AA
RS6732358	99,781,580	AA	AA	AA	AA	AB
RS6723474	99,799,700	BB	BB	BB	BB	BB
RS4621208	99,799,980	AA	AA	BB	AA	AA
RS7608476	99,801,970	AA	AA	AA	AA	AA
RS7601309	99,806,860	BB	BB	AA	BB	BB
RS1366765	99,807,780	BB	BB	AA	BB	BB
RS4481043	99,808,620	BB	BB	BB	BB	BB
RS17023158	99,814,360	AA	AA	AA	AA	AA
RS17023161	99,815,040	AB	AA	BB	BB	AB
RS6542901	99,815,130	BB	BB	BB	BB	BB
RS17023167	99,816,640	AA	AA	AA	AA	AA
RS10191077	99,817,970	BB	BB	BB	BB	BB
RS7586604	99,818,110	AA	AA	AA	AA	AA
RS1864273	99,820,520	AA	AA	BB	AA	AA
RS6731666	99,821,890	BB	BB	BB	BB	BB
RS12473053	99,823,460	BB	BB	BB	BB	BB
RS4850915	99,828,540	BB	BB	AA	BB	BB
RS6756248	99,830,620	AA	BB	AA	AA	AA
RS10865034	99,831,410	BB	BB	BB	BB	BB
RS11123800	99,831,460	BB	BB	AA	BB	BB

RS17023216	99,832,580	AA	BB	BB	AB	AB
RS4286327	99,835,380	AA	AA	AA	AA	AA
RS17023236	99,842,640	AA	AA	BB	AA	AA
RS6715454	99,847,380	BB	AB	BB	AA	AA
RS17023254	99,848,960	AA	AA	BB	AA	AA
RS17023256	99,856,580	AA	AA	AA	AA	AA
RS17023265	99,860,460	AA	AA	AA	AA	AA
RS7591889	99,860,500	AA	AA	AA	AA	AA
RS17436705	99,860,660	AA	AA	BB	AA	AA
RS11682684	99,868,140	AB	AA	AA	AA	AA
RS13423929	99,871,860	BB	BB	BB	BB	BB
RS6735012	99,882,570	AA	BB	AA	BB	BB
RS7593337	99,894,660	AA	BB	AA	BB	BB
RS6707538	99,911,380	AA	AA	AA	AA	AA
RS17023310	99,913,810	BB	BB	BB	BB	BB
RS7577040	99,921,380	BB	AA	BB	AB	AB
RS6708436	99,936,160	AA	AA	AA	AA	AA
RS13023088	99,942,740	BB	BB	AB	BB	BB
RS10187195	99,947,760	BB	AA	BB	AB	AB
RS10198187	99,947,980	AA	AA	AA	AB	AB
RS17023362	99,969,710	BB	BB	BB	AB	AB
RS17023404	99,985,700	BB	BB	BB	BB	BB
RS10205511	99,990,480	BB	AA	BB	BB	BB
RS17023424	99,996,180	AA	AA	AA	AA	AA
RS10198062	100,002,100	AA	AA	AA	AB	AB
RS11685341	100,003,200	BB	AA	BB	AB	AB
RS7605430	100,014,000	BB	AA	BB	AB	AB
RS11693242	100,029,300	AA	AA	AA	AA	AA
RS12712063	100,029,700	AA	AA	AA	AA	AA
RS13416841	100,030,000	BB	BB	BB	BB	BB
RS17352801	100,031,100	AA	BB	AA	AB	AB
RS2028137	100,032,100	AA	AA	AA	AA	AA
RS4851244	100,037,900	BB	BB	BB	BB	BB
RS735001	100,048,000	BB	BB	BB	BB	BB
RS17023468	100,060,500	AA	AA	AA	AA	AA
RS10177358	100,096,500	AA	AA	AA	AA	AA
RS6749757	100,096,500	BB	AA	BB	AB	AB
RS11694875	100,099,300	BB	AA	BB	AB	AB
RS17023500	100,100,900	BB	BB	BB	BB	BB
RS6745099	100,105,900	AA	AA	AA	AB	AB
RS17437538	100,106,200	BB	BB	BB	BB	BB
RS6706188	100,106,400	AA	AA	AA	AB	AB
RS2309750	100,108,200	AA	AA	AA	AB	AB
RS17023529	100,110,600	BB	BB	BB	BB	BB
RS17023532	100,112,300	BB	BB	BB	BB	BB
RS1561230	100,113,100	BB	AA	BB	AB	AB
RS11885475	100,113,300	AA	AA	AA	AA	AA
RS13000759	100,120,300	BB	BB	BB	BB	BB
RS13415595	100,123,400	AA	AA	AA	AA	AA
RS4851252	100,123,700	AA	AA	AA	AA	AA
RS11681966	100,125,900	BB	BB	BB	BB	BB

RS17023569	100,126,000	BB	BB	BB	BB	BB
RS17023571	100,126,300	AA	BB	AA	AA	AA
RS12712066	100,129,400	AA	BB	BB	AB	AB
RS10496344	100,130,600	BB	BB	BB	BB	BB
RS11123813	100,132,100	AA	AA	AA	AA	AA
RS4851256	100,141,500	AA	BB	AA	AA	AA
RS7593261	100,156,300	AA	BB	AA	AA	AA
RS11902629	100,161,500	AA	AA	AA	AA	AA
RS11902705	100,161,600	BB	AA	BB	BB	BB
RS4851262	100,162,700	AA	AA	AA	AA	AA
RS17023659	100,165,400	BB	BB	BB	BB	BB
RS4556997	100,181,300	BB	AA	AA	AA	AA
RS4851266	100,184,900	AA	AA	BB	AA	AA
RS13401183	100,186,100	AA	AA	AA	AA	AA
RS6542919	100,186,600	BB	BB	BB	BB	BB
RS9653442	100,191,800	AA	BB	AA	BB	BB
RS4851269	100,196,800	AA	AA	AA	BB	BB
RS1160542	100,198,600	AA	BB	AA	BB	BB
RS2871344	100,205,900	AA	AA	BB	AA	AA
RS13026283	100,209,100	AA	AA	BB	AA	AA
RS17023801	100,217,500	AA	BB	BB	BB	BB
RS10865036	100,227,000	BB	BB	BB	AA	AA
RS2309811	100,228,800	BB	BB	BB	AA	AA
RS12053126	100,231,100	AA	AA	BB	AA	AA
RS13026143	100,231,800	BB	AA	AA	BB	BB
RS6748892	100,232,500	AA	BB	BB	BB	BB
RS17023825	100,234,900	BB	AA	AA	AA	AA
RS17023838	100,236,800	BB	BB	BB	BB	BB
RS4438499	100,243,200	AA	BB	AA	BB	BB
RS6542922	100,245,300	AA	BB	AA	BB	BB
RS12467008	100,248,900	AA	AA	AA	AA	AA
RS10178929	100,250,200	BB	BB	BB	BB	BB
RS2871341	100,254,900	AA	AA	AA	AA	AA
RS4622752	100,258,400	BB	BB	AA	BB	BB
RS6542923	100,258,900	BB	BB	BB	BB	BB
RS6542924	100,259,500	AA	AA	AA	AA	AA
RS6727379	100,261,700	AA	AA	AA	AA	AA
RS2309819	100,266,100	AA	BB	BB	BB	BB
RS2309821	100,266,700	BB	BB	BB	BB	BB
RS2309823	100,268,000	AA	AA	AA	AA	AA
RS2309824	100,268,100	AA	AA	AA	AA	AA
RS13409545	100,278,800	BB	AA	BB	AA	AA
RS7582180	100,278,900	BB	AA	AA	AA	AA
RS10180423	100,285,400	BB	AA	BB	AA	AA
RS10207566	100,294,500	AA	AA	AA	AA	AA
RS11123826	100,302,700	BB	BB	AA	BB	BB
RS6757754	100,317,700	BB	AA	AA	BB	BB
RS2118468	100,319,000	BB	AA	BB	BB	BB
RS13031508	100,319,200	AA	AA	BB	AA	AA
RS17024021	100,322,200	BB	BB	BB	BB	BB
RS11686880	100,322,700	AA	AA	BB	AA	AA

RS6728575	100,330,400	BB	AA	AA	AA	AA
RS7598205	100,330,800	BB	AA	AA	AA	AA
RS17024035	100,335,000	AA	AB	BB	BB	BB
RS4850927	100,335,500	AA	AA	BB	AA	AA
RS17024054	100,337,000	AA	AA	AA	AA	AA
RS6738410	100,337,600	AA	AA	BB	BB	BB
RS13392198	100,342,100	BB	BB	AA	AA	AA
RS11685401	100,343,100	BB	BB	BB	AA	AA
RS4640404	100,347,000	AA	AA	AA	BB	BB
RS1437971	100,353,400	BB	BB	BB	BB	BB
RS10206374	100,357,300	AA	AA	BB	BB	BB
RS6542946	100,361,000	AA	AA	BB	BB	BB
RS17024129	100,361,600	AA	AA	BB	BB	BB
RS7585898	100,364,700	AA	AA	BB	BB	BB
RS6716367	100,366,400	AA	AA	BB	BB	BB
RS1370627	100,375,800	AA	BB	BB	BB	BB
RS12467104	100,379,500	BB	BB	BB	BB	BB
RS2099611	100,387,500	BB	BB	BB	AA	AA
RS4149513	100,389,200	AA	AA	BB	AA	AA
RS3791358	100,398,300	BB	BB	AA	AA	AA
RS6740922	100,414,500	BB	BB	BB	BB	BB
RS13416322	100,419,000	AA	BB	AA	BB	BB
RS4851316	100,419,200	AA	BB	AA	BB	BB
RS763962	100,419,900	BB	AA	BB	AA	AA
RS11695568	100,424,900	AA	AA	AA	AA	AA
RS7561516	100,425,600	BB	BB	BB	BB	BB
RS750562	100,430,000	BB	AA	BB	AA	AA
RS13383638	100,431,100	AA	BB	AA	BB	BB
RS13398213	100,431,500	BB	AA	BB	AA	AA
RS10204004	100,431,800	AA	BB	AA	BB	BB
RS6717716	100,433,100	AA	AA	AA	BB	BB
RS17024335	100,433,400	AA	BB	AA	AA	AA
RS962556	100,434,200	BB	BB	BB	AA	AB
RS13411940	100,453,400	BB	AA	BB	BB	BB
RS17024368	100,454,000	BB	AA	BB	BB	BB
RS1519661	100,454,700	AA	BB	AA	AA	AA
RS753439	100,455,300	AA	BB	AA	AA	AA
RS11123833	100,457,600	BB	AA	BB	BB	BB
RS7568283	100,463,000	AA	BB	AA	AA	AA
RS13428749	100,463,300	AA	BB	AA	AA	AA
RS7568519	100,463,300	BB	AA	BB	BB	BB
RS13393776	100,463,300	BB	AA	BB	BB	BB
RS11123834	100,472,100	AA	BB	AA	AA	AA
RS11900367	100,473,600	AA	BB	AA	AA	AA
RS1000867	100,476,200	BB	BB	BB	BB	BB
RS13389527	100,478,600	AA	BB	AA	AA	AA
RS17024434	100,478,900	BB	BB	BB	BB	BB
RS1370624	100,480,100	AA	AA	AA	AA	AA
RS17024446	100,481,100	BB	AA	BB	AA	AA
RS964559	100,488,200	BB	BB	BB	AA	AA
RS12614705	100,489,200	AA	AA	AA	AA	AA

RS6718243	100,494,000	AA	AA	AA	AA	AA
RS17024471	100,505,000	BB	BB	BB	BB	BB
RS17024473	100,505,500	BB	BB	BB	BB	BB
RS17024474	100,506,000	BB	BB	BB	BB	BB
RS4147028	100,509,500	BB	BB	AA	BB	BB
RS2176733	100,511,900	BB	BB	BB	AA	AA
RS12474526	100,516,000	BB	AA	AA	BB	BB
RS7423870	100,518,800	AA	AA	BB	BB	BB
RS4851329	100,526,200	BB	AA	AA	AB	AB
RS1533655	100,531,200	BB	AA	AA	AB	AB
RS6753993	100,539,600	BB	AA	AB	AB	AB
RS2139403	100,544,800	AA	AA	AA	AB	AB
RS17640099	100,549,700	BB	BB	BB	BB	BB
RS2971007	100,550,000	AA	BB	BB	AB	AB
RS12469806	100,558,400	AA	AA	AA	AA	AA
RS2309846	100,567,900	AA	AA	BB	AB	AB
RS7594990	100,568,100	AA	AA	AA	AA	AA
RS4851346	100,589,800	BB	AA	AA	AB	AB
RS10178303	100,591,700	AA	AA	AA	AA	AA
RS2971010	100,593,000	BB	BB	BB	AB	AB
RS13386350	100,606,100	AA	AA	BB	BB	BB
RS1533657	100,607,800	AA	AA	AA	AA	AA
RS2139405	100,608,800	BB	BB	AA	AA	AA
RS2946601	100,609,100	BB	BB	AA	AA	AA
RS2222282	100,611,200	AA	AA	AA	AA	AA
RS10191620	100,613,600	BB	BB	AA	AA	AA
RS6738852	100,614,800	AA	AA	BB	BB	BB
RS10187537	100,617,200	BB	BB	AB	AA	AA
RS4599142	100,620,700	BB	BB	AA	AA	AA
RS13402095	100,620,900	AA	AA	AA	AA	AA
RS11695640	100,630,800	AA	AA	AA	AA	AA
RS10153955	100,631,300	AA	AA	BB	BB	BB
RS4850942	100,641,300	AB	AA	BB	BB	BB
RS4851359	100,650,000	AA	AA	AA	AA	AA
RS10201136	100,652,500	AA	AA	AA	AA	AA
RS4851361	100,652,700	AA	AA	BB	BB	BB
RS4850944	100,653,300	AA	AA	AA	AA	AA
RS17024601	100,657,500	BB	BB	AA	AA	AA
RS4851365	100,657,600	AA	AA	BB	BB	BB
RS10209092	100,658,500	BB	BB	AA	AA	AA
RS3112238	100,662,500	BB	BB	BB	BB	BB
RS2942894	100,663,400	AA	AA	AA	AA	AA
RS2942895	100,663,600	BB	BB	BB	BB	BB
RS2971024	100,663,900	AA	AA	AA	AA	AA
RS2942905	100,674,700	BB	BB	BB	BB	BB
RS2942909	100,676,500	AA	AA	AA	AA	AA
RS1398867	100,676,700	AA	AA	AA	AA	AA
RS6735550	100,677,500	BB	BB	BB	BB	BB
RS3112252	100,678,500	AB	AB	AB	AB	AB
RS1355819	100,678,900	AA	AA	AA	AA	AA
RS1355820	100,679,000	AA	AA	AA	AA	AA

RS2942879	100,682,700	BB	BB	BB	BB	BB
RS3112256	100,688,300	AA	AA	BB	AB	AB
RS6729336	100,688,600	AA	AA	AA	AA	AA
RS2970991	100,688,600	AA	AA	BB	AB	AB
RS2942884	100,690,000	BB	BB	AA	AB	AB
RS7558261	100,691,100	BB	BB	AA	AB	AB
RS13384306	100,691,600	BB	BB	BB	BB	BB
RS11901892	100,693,000	BB	BB	BB	BB	BB
RS3112257	100,693,300	BB	BB	AA	AB	AB
RS12615536	100,695,600	BB	BB	AA	AB	AB
RS11123842	100,704,400	AA	AA	AB	AB	AB
RS11123843	100,704,500	BB	BB	AA	AB	AB
RS4851370	100,704,900	AA	AA	BB	AB	AB
RS17024677	100,706,900	AA	AA	AA	AA	AA
RS907794	100,708,000	BB	BB	AA	BB	BB
RS1567803	100,709,400	BB	AA	AA	AB	AB
RS12621996	100,709,800	BB	AA	AA	AB	AB
RS1567804	100,710,100	BB	AA	AA	AB	AB
RS1567805	100,710,100	AA	BB	BB	AB	AB
RS6708706	100,724,300	BB	BB	BB	BB	BB
RS12465343	100,736,900	AA	AA	AA	AA	AA
RS1107634	100,745,400	BB	AA	BB	BB	BB
RS17024707	100,747,200	BB	BB	BB	BB	BB
RS6746018	100,747,300	BB	AA	BB	BB	BB
RS10172286	100,750,400	BB	AA	BB	BB	BB
RS6741485	100,754,700	BB	BB	BB	BB	BB
RS10177037	100,760,100	BB	BB	BB	BB	BB
RS17024737	100,762,000	BB	BB	BB	BB	BB
RS12467539	100,768,800	AA	AA	AA	AA	AA
RS10198664	100,773,600	BB	AA	BB	BB	BB
RS7577100	100,776,000	BB	BB	BB	BB	BB
RS7565981	100,790,800	BB	BB	BB	BB	BB
RS11893548	100,795,200	BB	AA	BB	BB	BB
RS10172146	100,800,700	BB	BB	BB	BB	BB
RS6542993	100,807,100	AA	AA	AA	AA	AA
RS13028398	100,807,300	AA	AA	AA	AB	AB
RS4349369	100,810,200	AA	AA	AA	AA	AA
RS17024852	100,816,300	AA	AA	AA	AA	AA
RS3860456	100,816,700	BB	BB	BB	BB	BB
RS3888170	100,817,100	AA	AA	AA	AA	AA
RS7587153	100,818,900	BB	BB	BB	BB	BB
RS7587470	100,819,200	BB	BB	BB	AB	AB
RS7587573	100,819,300	AA	AA	AA	AA	AA
RS7582455	100,838,100	BB	BB	BB	AA	AA
RS2043534	100,847,300	BB	AA	BB	AA	AB
RS6542996	100,852,300	AA	BB	AA	BB	BB
RS12472319	100,853,900	BB	AA	BB	AA	AA
RS13387032	100,857,900	BB	BB	BB	BB	BB
RS13390036	100,858,100	BB	BB	BB	BB	BB
RS2164319	100,862,500	AA	AA	AA	AB	AB
RS13012765	100,864,600	AA	BB	AA	BB	BB

RS7595380	100,869,200	BB	BB	BB	BB	BB
RS1369482	100,869,800	AA	AA	AA	AA	AA
RS1369481	100,878,400	AA	BB	BB	AA	AA
RS17654748	100,878,900	BB	AA	BB	BB	BB
RS17654772	100,879,000	AA	AA	AA	AA	AA
RS10194413	100,885,300	BB	BB	BB	AB	AB
RS4851377	100,888,700	AA	BB	BB	AB	AB
RS12479086	100,890,000	BB	BB	BB	BB	BB
RS13394520	100,890,200	AA	BB	BB	BB	BB
RS4851379	100,890,700	AA	AA	AA	AB	AB
RS7570190	100,894,500	AA	AA	AA	AA	AA
RS11674168	100,895,600	AA	AA	AA	AA	AA
RS11676107	100,902,900	AA	AA	AA	AB	AB
RS13026599	100,907,700	AA	AA	BB	AA	AA
RS17717414	100,913,000	AA	BB	BB	BB	BB
RS12466423	100,913,500	BB	BB	BB	BB	BB
RS3754675	100,916,300	BB	BB	BB	BB	BB
RS17025005	100,920,000	AA	AA	AA	AA	AA
RS6542999	100,920,200	AA	AA	AA	AA	AA
RS13010122	100,926,500	BB	BB	AA	AA	AA
RS4851384	100,928,100	AA	AA	AA	AA	AA
RS10170955	100,928,300	BB	BB	BB	BB	BB
RS10206435	100,928,900	BB	BB	AA	AB	AA
RS12612424	100,937,000	AA	AA	AA	AA	AA
RS882272	100,942,600	AA	AA	BB	BB	BB
RS4851391	100,946,200	AA	AA	AA	AA	AA
RS9679638	100,960,900	AA	AA	AA	AA	AA
RS17025130	100,961,300	AA	AA	AA	AA	AA
RS1542178	100,961,900	AA	AA	BB	BB	BB
RS2278727	100,965,100	BB	AA	BB	BB	BB
RS1053096	100,979,000	BB	AA	AA	BB	BB
RS10189697	100,983,900	BB	BB	BB	AB	AB
RS2278723	100,985,800	BB	AA	AA	BB	BB
RS17025161	100,985,800	AA	AA	AA	AA	AA
RS2278721	100,987,400	BB	BB	BB	BB	BB
RS17025170	100,988,100	BB	BB	BB	BB	BB
RS17663443	101,003,400	AB	AB	AB	BB	BB
RS3739014	101,005,300	AA	BB	BB	AA	AA
RS12474501	101,009,300	BB	AA	BB	BB	BB
RS13412722	101,013,200	BB	BB	BB	AB	AB
RS11123859	101,032,300	BB	BB	BB	AA	AA
RS17025231	101,043,100	BB	BB	BB	BB	BB
RS10496351	101,046,600	AA	BB	AA	AA	AA
RS2121367	101,049,900	BB	AA	AA	AA	AA
RS6543013	101,057,600	BB	BB	AA	BB	BB
RS4851400	101,063,100	BB	BB	BB	BB	BB
RS6727200	101,064,600	BB	BB	AA	BB	BB
RS11678957	101,065,600	AA	AA	AA	AB	AB
RS2165428	101,072,400	BB	BB	BB	AB	AB
RS2871392	101,082,800	BB	BB	BB	AB	AB
RS2592392	101,089,800	BB	BB	AA	AA	AA

RS812717	101,094,900	AA	AA	AB	AA	AA
RS6543018	101,096,700	BB	BB	AA	AA	AA
RS7559325	101,097,000	AA	AA	AA	AB	AB
RS2016986	101,106,100	BB	AA	AA	BB	BB
RS935066	101,106,500	AA	AA	AA	AB	AB
RS732136	101,106,700	BB	BB	BB	AB	AB
RS12233027	101,107,700	BB	BB	BB	BB	BB
RS12472431	101,116,200	AA	AA	AA	AA	AA
RS11686264	101,116,400	AA	AA	AA	AB	AB
RS13412911	101,121,800	AA	BB	AA	AA	AA
RS7604060	101,126,300	BB	BB	BB	BB	BB
RS11691031	101,145,800	BB	AA	BB	AA	AA
RS1620893	101,163,200	AA	BB	AA	BB	BB
RS4850962	101,168,100	AA	AA	AA	AB	AB
RS6543023	101,182,400	AA	AA	AA	AA	AA
RS7603503	101,186,100	AA	AA	AA	AA	AA
RS13408291	101,214,700	AA	AA	AA	AA	AA
RS1660702	101,232,400	AA	BB	BB	BB	BB
RS10182839	101,243,900	BB	BB	BB	BB	BB
RS750783	101,255,700	BB	BB	AB	AB	BB
RS1192793	101,264,300	AB	BB	BB	BB	BB
RS17025374	101,282,700	AB	BB	BB	BB	BB
RS13031025	101,283,900	AB	AA	BB	AB	AB
RS17025378	101,286,000	AB	BB	BB	BB	BB
RS17190412	101,289,200	AB	BB	AA	AB	AB
RS2056716	101,295,400	AB	BB	AA	AA	AA
RS4851420	101,322,600	AA	AA	AA	AA	AA
RS4539813	101,331,800	BB	BB	BB	BB	BB
RS6749888	101,337,300	BB	BB	BB	BB	BB
RS6735216	101,337,400	AA	AA	AA	AA	AA
RS6753727	101,338,000	AA	AA	AA	AB	AB
RS11674064	101,350,500	AB	AA	AA	AA	AA
RS4850969	101,352,200	AB	AA	AA	AA	AA
RS3923053	101,352,800	BB	BB	BB	AB	AB
RS7572740	101,354,900	AA	AA	AA	AA	AA
RS7584951	101,361,200	AB	BB	BB	BB	BB
RS9308846	101,364,200	AB	AA	AA	AB	AB
RS4069937	101,377,400	AA	BB	AA	AA	AA
RS6716306	101,381,200	BB	AA	BB	AB	AB
RS4241206	101,383,500	BB	AA	BB	AB	AB
RS11689348	101,395,700	BB	BB	BB	AB	AB
RS12620464	101,396,100	AA	BB	AA	AA	AA
RS908136	101,398,200	AA	AA	AA	AA	AA
RS7607798	101,409,500	AA	AA	AA	AA	AA
RS6543046	101,409,700	BB	BB	BB	BB	BB
RS2037083	101,413,700	BB	AA	BB	BB	BB
RS17025611	101,418,300	AA	AA	AA	AA	AA
RS938301	101,419,600	BB	AA	BB	BB	BB
RS6705262	101,421,500	AA	BB	AA	AA	AA
RS11123880	101,422,900	AA	BB	AA	AA	AA
RS7583866	101,431,200	BB	BB	BB	AB	AB

RS12712105	101,431,700	AA	BB	AA	AB	AB
RS4850977	101,432,400	BB	AA	BB	BB	BB
RS4850978	101,432,400	BB	AA	BB	BB	BB
RS4851454	101,447,000	BB	BB	BB	AB	AB
RS6720584	101,447,400	AB	BB	AA	AA	AA
RS7574003	101,447,600	AB	BB	BB	BB	BB
RS6707298	101,456,200	AB	BB	BB	BB	BB
RS11694135	101,472,000	AA	AA	AA	AA	AA
RS9653469	101,476,000	BB	BB	BB	BB	BB
RS17025718	101,482,200	BB	BB	BB	BB	BB
RS1509493	101,489,200	AA	AA	AA	AA	AA
RS1995826	101,495,900	AB	AA	BB	AB	AB
RS10206804	101,498,900	AB	AA	BB	AB	AB
RS1848699	101,504,300	AB	BB	AA	AB	AB
RS1509497	101,504,600	AB	BB	AA	AB	BB
RS3106053	101,509,800	BB	BB	BB	BB	BB
RS6745920	101,512,100	BB	BB	BB	BB	BB
RS290775	101,521,600	AA	AA	AA	AA	AA
RS6543072	101,522,700	BB	BB	BB	AB	AB
RS4308140	101,533,300	AA	AA	AA	AB	AB
RS17195147	101,534,300	BB	BB	BB	BB	BB
RS17025782	101,534,400	AA	AA	AA	AA	AA
RS6543074	101,534,400	BB	BB	BB	AB	AB
RS7585029	101,543,400	AB	BB	BB	AB	AB
RS7592857	101,545,700	AB	BB	AA	AB	AB
RS13420057	101,552,200	AA	AA	AA	AA	AA
RS6725026	101,557,200	AB	AA	BB	AA	AA
RS6543079	101,562,900	AB	BB	BB	AB	AB
RS7355302	101,592,400	AB	BB	AA	AB	AB
RS10197455	101,594,000	BB	BB	BB	AB	AB
RS17025912	101,614,500	AA	AA	AA	AA	AA
RS878203	101,618,100	AA	AA	AA	AA	AA
RS1018218	101,618,400	BB	BB	BB	BB	BB
RS1018219	101,618,400	BB	BB	BB	BB	BB
RS4851475	101,628,000	AB	AA	BB	AB	AB
RS951796	101,636,800	BB	BB	BB	BB	BB
RS10202056	101,640,800	AA	AA	AA	AA	AA
RS7598866	101,641,000	AB	AA	AA	AA	AA
RS10194088	101,641,000	AB	AA	BB	BB	BB
RS10496359	101,657,900	AA	AA	AA	AA	AA
RS2016677	101,658,700	AA	AA	AA	BB	BB
RS6543085	101,661,300	AB	AA	BB	BB	BB
RS13396452	101,677,500	AA	AA	AA	AA	AA
RS17025961	101,687,000	AA	AA	AA	AA	AA
RS7597661	101,717,500	BB	AB	BB	BB	BB
RS17026045	101,753,500	BB	BB	BB	BB	BB
RS17026058	101,764,000	BB	BB	BB	AB	AB
RS17026061	101,764,100	BB	BB	BB	AA	AB
RS6733385	101,773,800	AA	AA	AA	AB	AB
RS6543095	101,779,000	BB	AA	BB	AB	AB
RS4851494	101,781,700	BB	BB	BB	AB	AB

RS12329012	101,794,900	BB	BB	BB	BB	BB
RS2236936	101,810,300	AA	AA	AA	AB	AB
RS7599763	101,824,000	AA	AA	AA	AA	AA
RS17802002	101,829,000	AB	AA	AA	AA	AA
RS2158816	101,834,100	AA	AA	AA	AB	AB
RS13425874	101,844,800	BB	AA	BB	BB	BB
RS3771903	101,845,500	BB	BB	BB	AB	AA
RS9967668	101,854,300	BB	BB	BB	AB	AB
RS7574674	101,872,100	BB	AA	BB	AB	AB
RS17746734	101,904,200	AB	AA	BB	AA	AB
RS2214890	101,909,100	BB	AA	BB	AB	AB
RS12373806	101,909,800	AB	AA	BB	AA	AA
RS12990046	101,909,900	AA	AA	AA	AA	AA
RS11688863	101,917,300	AB	BB	AA	AB	AB
RS12185627	101,927,200	AB	BB	AA	AB	AB
RS4624399	101,928,200	BB	BB	BB	BB	BB
RS10865049	101,937,500	AB	BB	BB	BB	BB
RS1859716	101,939,900	AA	AB	AA	AB	AB
